# Comparing outcomes of COVID-19 and influenza among hospitalized adults in the European severe acute respiratory infection vaccine effectiveness (EuroSAVE) network, 2021–2024: a retrospective cohort analysis

**DOI:** 10.1016/j.lanepe.2026.101672

**Published:** 2026-04-16

**Authors:** Amy Gimma, Kujtim Mersini, Olgha Tarkhan-Mouravi, Besfort Kryeziu, Kristina Stavridis, Dinagul Otorbaeva, Najada Como, Khatuna Zhakhashvili, Ariana Kalaveshi, Elizabeta Jancheska, Adyl Ryspaev, Sandra Cohuet, James Humphreys, Angela M.C. Rose, James E. Fielding, Piers Mook, Marc-Alain Widdowson, Oksana Artemchuk, Iris Finci, Mark A. Katz

**Affiliations:** a WHO Regional Office for Europe, Denmark; b Institute of Public Health, Albania; c National Center for Disease Control and Public Health, Georgia; d National Institute of Public Health, Kosovo; e Institute of Public Health of the Republic of North Macedonia, North Macedonia; f Department for Disease Prevention and State Sanitary and Epidemiological Surveillance, Kyrgyzstan; g Medical University of Tirana, Albania; h Laboratory of Molecular Genetic Research, Kyrgyzstan; i Epiconcept, France

**Keywords:** Respiratory virus, SARI, COVID-19, SARS-CoV-2, Influenza, Severity, Hospital outcome, ICU, Death, EuroSAVE, MIC, Middle Income Countries, Albania, Georgia, Kyrgyzstan, North Macedonia, Kosovo, Eastern Europe, WHO Euro, World Health Organization Regional Office for Europe

## Abstract

**Background:**

Understanding changes in the severity of respiratory virus infections over time is critical for guiding public health interventions. Few studies have been conducted in middle-income countries (MICs), where healthcare infrastructure, COVID-19 and influenza vaccine coverage, and antiviral availability and use differ from high-income countries.

**Methods:**

We compared the odds of severe outcome and death by virus test result among patients ≥18 years old hospitalized with severe acute respiratory infection (SARI) in five MICs and areas (Albania, Georgia, Kyrgyzstan, North Macedonia, and Kosovo). Severe outcome included ICU admission, mechanical ventilation, ECMO, or death during hospitalization. We calculated adjusted odds ratios (aORs) and 95% confidence intervals (CIs) for severe outcome and death by respiratory virus PCR test result. Influenza-positive patients were the reference group. We grouped SARS-CoV-2 patients into three periods based on COVID-19 activity and circulating Omicron subvariants [Period 1 (6 December 2021–18 December 2022), Period 2 (19 December 2022–9 July 2023), and Period 3 (10 July 2023–6 August 2024).] We adjusted for age, sex, comorbidities, COVID-19 vaccination status, and country.

**Findings:**

Of 7671 patients who met the inclusion criteria, 963 (13%) patients had a severe outcome; 749 (10%) were admitted to ICU, 202 (3%) received mechanical ventilation or ECMO and 285 (4%) died. Compared to influenza patients, SARS-CoV-2-positive patients had a higher odds of severe outcome in Period 1 [ aOR = 1.80 (95% CI 1.23–2.64)], P2 [aOR = 1.94 (95% CI 1.25–3.01)], and Period 3 [aOR = 1.63 (95% CI 1.05–2.54)]. COVID-19 patients in Period 1 (aOR = 2.80; 1.67–4.82) and Period 3 (aOR = 2.25; 95% CI 1.23–4.18) had a higher aOR for death compared to influenza-positive patients; for Period 2 the aOR was 1.04 (95% CI 0.48–2.15).

**Interpretation:**

The odds of severe outcome were higher for SARS-CoV-2 patients compared to influenza-positive patients for all periods, while the odds of death were higher during Period 1 and Period 3. These results underscore the continued severity of COVID-19, and need for COVID-19-related health interventions, including COVID-19 vaccination and in-hospital patient management.

**Funding:**

This study was funded by the World Health Organization/Regional Office for Europe, in part through a cooperative agreement with the U.S. Centers for Disease Control and Prevention.

**Research in context**

**Evidence before this study**

Understanding changes in the severity of respiratory virus infections over time, particularly with respect to new viruses like SARS-CoV-2, is critical for guiding public health interventions. Understanding the changing severity of a novel virus relative to known respiratory viral infections, such as influenza, can help provide context using a comparator for which severity has been widely described. We searched PubMed and Embase for studies comparing in-hospital severity of COVID-19 and influenza published between 1 January 2022 and 15 August 2024, using the search terms (“COVID-19” OR “SARS-CoV-2”) AND (“influenza”) AND (“hospital∗” OR “in-hospital”) AND (“severity” OR “outcome” OR “ICU” OR “death”), without language restrictions. We included studies comparing mortality and severe outcomes (e.g., ICU admission, mechanical ventilation) between adult COVID-19 and influenza patients. Several studies from high-income countries reported higher risks of ICU admission, mechanical ventilation, and mortality in COVID-19 patients compared to those with influenza from the beginning of the pandemic, in 2020, through 2024. Some studies found a decline in relative severity of COVID-19 compared to influenza over time, potentially due to increasing population immunity and the emergence of less virulent SARS-CoV-2 variants. There were few studies from low- and middle-income countries (LMICs), where healthcare infrastructure, COVID-19 and influenza vaccine coverage, and antiviral availability and use differ from high-income countries.

**Added value of this study**

To our knowledge, this is the first multicountry analysis from middle-income countries in Europe to compare the severity of in-hospital outcomes among adult patients ≥18 years old with COVID-19 and influenza during the Omicron period (2021–2024). Using standardized SARI surveillance protocols across five countries and areas, we found that COVID-19 was associated with significantly higher odds of ICU admission, mechanical ventilation, and in-hospital death compared to influenza, even in the period following the end of the COVID-related Public Health Event of International Concern (May 2023). Our study also documented concerningly low COVID-19 vaccine uptake among hospitalized patients, many of whom were at high risk for severe COVID-19 disease. We also documented low influenza vaccine uptake in these same patients who were at high risk for severe outcomes from influenza.

**Implications of all the available evidence**

COVID-19 continues to result in more severe outcomes than influenza among hospitalized adults in middle-income countries in the eastern part of the WHO European Region. These findings underscore the need to promote up-to-date COVID-19 vaccination among high-risk individuals and highlight the importance of vigilant in-hospital management, including antiviral therapies, to reduce severe outcomes from COVID-19.

## Introduction

Since the World Health Organization (WHO) declared an end to the Public Health Emergency of International Concern (PHEIC) for coronavirus disease (COVID-19) in May 2023,^1^ the severe acute respiratory syndrome coronavirus 2 (SARS-CoV-2) virus has continued to cause severe disease and deaths, contributing to strain on hospital systems.^2^

Understanding the severity of COVID-19 disease over time is critical to guide clinical management and inform public health programs such as vaccination.^3^^,^^4^ In addition, understanding COVID-19 disease severity can direct hospital planning and resource allocation, particularly in winter months, when hospitalizations due to other respiratory viruses, including influenza and respiratory syncytial virus (RSV), continue to cause a considerable burden.^5^

During the 3 years of the COVID-19 pandemic (2020–2023), COVID-19 resulted in significantly more in-hospital severe outcomes compared to influenza.^6^ However, studies published between 2023 and 2025 suggest that the severity of COVID-19 disease may be decreasing relative to the severity of disease caused by other respiratory viruses, such as influenza.^7^, ^8^, ^9^, ^10^, ^11^

Most research on COVID-19 severity using in-hospital severity of influenza as a reference has been conducted in a limited number of high-income countries.^6^, ^7^, ^8^, ^9^, ^10^, ^11^, ^12^ In middle-income countries (MICs), resources, including antiviral medications, are more limited, and COVID-19 vaccination accessibility and coverage has generally been lower compared to high-income countries.^13^ In addition, healthcare access, population demographics, and comorbidity profiles vary.^14^, ^15^, ^16^ As a result, in-hospital outcomes among COVID-19 patients may be different.

We aimed to compare the odds of in-hospital severe outcomes among hospitalized SARS-CoV-2-positive severe acute respiratory infection (SARI) patients with those of hospitalized influenza-positive SARI patients during 2021–2024 in five middle-income countries and areas in the eastern part of the WHO European Region. We used influenza-positive SARI patients as a comparison for COVID-19-positive SARI patients because this comparator has been used in previous studies,^6^, ^7^, ^8^^,^^10^^,^^11^ and using it as a comparator allowed us to compare our findings in middle-income countries in the WHO European Region to findings of previous studies performed in high-income countries.^6^, ^7^, ^8^, ^9^, ^10^, ^11^ Furthermore, because influenza is a well-known respiratory infection for which the burden of hospitalization and severe outcomes have been widely described, it provides a familiar benchmark for comparison.

In addition to the above analysis, we identified demographic and clinical risk factors associated with severe outcomes among all SARI patients.

## Methods

### Study setting

We conducted our retrospective cohort analysis using data from SARI sentinel surveillance sites in five countries and areas involved in the European SARI vaccine effectiveness (EuroSAVE) network, a network of hospitals conducting SARI surveillance that was originally established to evaluate COVID-19 and influenza vaccine effectiveness (VE) among adult SARI patients.^3^ Our analysis included four upper middle-income countries and areas (Albania, Georgia, North Macedonia, and Kosovo^17^) and one lower MIC (Kyrgyzstan).

A total of 25 pre-existing national SARI sentinel surveillance hospitals were included in Albania (N = 8), Georgia (N = 5), Kyrgyzstan (N = 4), North Macedonia (N = 4), and Kosovo^17^ (N = 4).

Countries began contributing data to this analysis at different times: Albania (June 2022), Georgia (January 2023), Kyrgyzstan (April 2022), North Macedonia (February 2022), Kosovo^17^ (December 2021). All countries contributed cases through 6 August 2024.

### Data collection

Methods related to patient recruitment, testing, and data collection in EuroSAVE hospitals have been described previously.^3^ Briefly, at all sites, we recruited individuals ≥18 years old who met the WHO case definition for SARI, defined as an acute respiratory infection with history of fever or measured fever of ≥38C°, cough, and symptom onset within the last 10 days, and were hospitalized for at least 24 h.^18^ SARI patients were recruited systematically in all hospitals, according to preapproved protocols. We limited our data to patients recruited from 6 December 2021 to 6 August 2024.

At all sites, hospital staff administered a brief questionnaire to all SARI patients. Questions addressed demographics, comorbidities, including cancer, diabetes, heart disease, lung disease, asthma, neurological conditions, renal disease, and rheumatological conditions, history of present illness, and COVID-19 and influenza vaccination histories.

Nasopharyngeal or nasal specimens were collected from SARI patients. The type of swab collected depended on the Ministry of Health guidelines in the respective country and area of the hospital. Samples were tested for SARS-CoV-2 and influenza viruses by reverse transcription polymerase chain reaction (RT-PCR). Hospital staff collected additional information about patients’ hospital course, including whether patients received supplemental oxygen, were admitted to an intensive care unit (ICU), received mechanical ventilation, and were discharged, transferred to another hospital, or died in-hospital in all 25 hospitals.

All countries had electronic COVID-19 vaccine registries, but not all countries had electronic influenza vaccine registries. Study staff verified participants’ COVID-19 vaccination history through national electronic immunization registries. Influenza vaccination history was collected through national electronic registries when possible; otherwise, influenza vaccination status was collected through self-report.

### Study outcomes and definitions

We evaluated two in-hospital outcomes for SARI patients: 1) death and 2) severe outcome, a composite variable that included, mechanical ventilation, extracorporeal membrane oxygenation (ECMO), admission to the intensive care unit (ICU), and/or death. For both outcomes, we only considered deaths that occurred during the current hospital admission, regardless of time since admission. In order to adjust for changes in calendar time that reflected changes in peak activity and hospital demand, we added a variable indicating whether the SARI admission occurred during a period of high activity to the main model. We considered periods of high activity to be months when SARI cases were in the highest quartile, which we determined by country.

We grouped patients into the following age categories: 18–59, 60–69, 70–79, and ≥80 years. These categories were selected to reduce confounding by age and to align with age categories commonly used in similar studies.

### Exclusion criteria

We limited our data to patients who had a PCR test that occurred within 12 days of symptom onset, in order to allow for patients who had symptom onset 10 days prior to hospital admission, and were swabbed within a maximum of 2 days of hospital admission. We excluded the following records from the analysis: 1) patients who did not meet the WHO SARI case definition; 2) patients who tested positive for both influenza and COVID-19; 3) patients with incomplete data for the severe outcome composite variable; 4) patients with incomplete demographic or comorbidity data; and 5) patients who were still hospitalized or had been transferred to another facility. In our main analysis, while we excluded patients with missing COVID-19 vaccination data, we did not exclude patients with missing influenza vaccination data, as influenza vaccine was not included in the model.

### Data curation

**Fig. 1:**
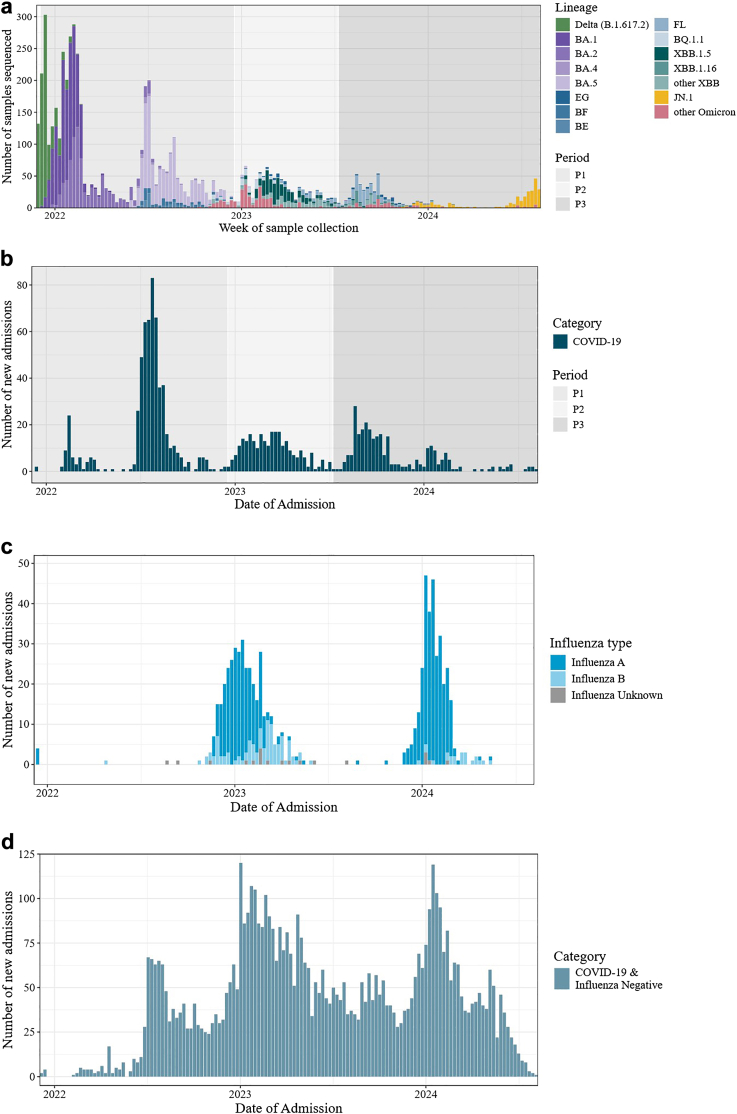
a) Number of SARS-CoV-2 samples by lineage for each week in the study period (6 December 2021–6 August 2023). Data are from all SARS-CoV-2 samples that were submitted to GISAID from the five countries and areas included in the analysis; b) COVID-19 cases by hospital admission date during the full study period (6 December 2021–6 August 2023). COVID-19 periods are indicated by background color; c) influenza cases by hospital admission date and influenza type (6 December 2021–6 August 2024); d) Test-negative SARI cases by hospital admission date (6 December 2021–6 August 2024).

In order to understand whether relative in-hospital severity of COVID-19 patients changed over time, we established three separate time periods for COVID patients during the study period (6 December 2021–6 August 2024, after applying exclusion criteria). We defined the three COVID-19 periods based on the distribution of the dates of admission of COVID-19 cases. We identified separate waves of COVID-19 that reflected changes in the magnitude of the weekly number of COVID-19 cases over time combined with changes in the predominance of variants from GISAID data in the five countries and areas (Fig. 1 A). We did not use specific case thresholds to determine the periods. We ultimately included three separate COVID-19 periods characterized by different predominant variants or subvariants and epidemiologic peaks.

Of the 1138 COVID-19 cases, 582 (51%) occurred in Period 1 (6 December 2021–18 December 2022), 266 (23%) in Period 2 (19 December 2022–9 July 2023), and 290 (25%) in Period 3 (10 July 2023–6 August 2024). We combined the two first waves into Period 1 because they were both characterized by the predominance of Delta and Omicron BA.1-5 activity. We combined multiple waves into Period 3 because these waves were associated with mixed Omicron subvariants and lower overall peaks.

In contrast to our approach for SARS-CoV-2, where we evaluated three separate periods during the two-year study period, we considered outcomes for influenza-positive and SARS-CoV-2- and influenza-negative patients SARI patients for the entire time period of the study.

### Main statistical analysis

We summarized categorical variables using counts and percentages, and continuous variables using medians and interquartile ranges. We assessed group differences for each variable using chi-squared or Fisher’s exact tests, as appropriate. We considered p-values < 0.05 statistically significant.

We evaluated the crude relationships between PCR test result groups, age, sex, comorbidities, and vaccination status with the outcomes of interest using univariable logistic regression.

Next, we defined five separate groups for analysis: patients who tested positive for influenza, patients who tested positive for COVID-19 for each of the three previously described waves, and patients who tested negative for both influenza and COVID-19. We fitted a multivariable logistic regression model to calculate the adjusted odds ratio (aOR) of the two outcomes for COVID-19-positive SARI patients in the three periods and SARS-CoV-2- and influenza-negative patients compared to the reference group of influenza-positive patients. The model also included age group, sex, comorbidities, COVID-19 vaccination status within the last 365 days, regardless of the total number of doses the patient received, and country of hospital admission. We selected all covariates based on clinical relevance; age, sex, and certain comorbidities have been identified as potential risk factors for severe outcomes from COVID and/or influenza infection.^19^, ^20^, ^21^ We did not include influenza vaccination in the model due to low influenza vaccination rates and incomplete influenza vaccination data. We present unadjusted ORs and aORs with 95% confidence interval (95% CI) and p-values.

### Secondary and sensitivity analyses

As a secondary analysis, we assessed differences in severity among influenza-positive patients by season. We defined each season as lasting from week 40 of one year through week 39 of the following year. We adjusted for the same variables as in the primary analysis, except for COVID-19 vaccination status. Unlike in the primary analysis, for this analysis we used a composite comorbidity variable, where anyone with at least one comorbidity was considered to have a comorbidity.

Additionally, we conducted a stratified analysis of individuals aged ≥60 years old and those 18–59 years old using the same methodology that we used for the primary analysis.

We also conducted a number of sensitivity analyses. First, we conducted an analysis that included only individuals recommended for influenza vaccine by WHO (adults ≥60 years old and adults aged 18–59 with at least one comorbidity).^22^

Second, because the effectiveness of COVID-19 vaccine has been shown to wane beyond 4–6 months,^3^ we conducted the same analysis as our primary analysis but only defined patients who had received a vaccine within 6 months prior to their SARI admission as vaccinated against COVID-19.

Third, we conducted a sensitivity analysis in which we included age as a continuous variable.

We conducted a fourth sensitivity analysis where we accounted for the nested structure of the data; we included 2 levels with a random intercept for hospital and a fixed effect for country.

In order to conduct the analysis without the potential confounders of vaccination, we conducted a fifth sensitivity analysis where we removed SARI patients who had been recently vaccinated against COVID-19 (<365 days) or vaccinated in the current season against influenza.

As another sensitivity analysis, in order to address potential biases caused by the small sample size, we conducted secondary analysis where we adjusted for differences between PCR test-result groups using inverse probability weighting (IPW). We estimated generalized propensity scores by fitting a multinomial logistic regression model with the PCR test-result group as the outcome and the variables used in the main model as predictors. We calculated stabilized weights as the marginal probability of each group divided by the individual’s predicted probability truncated at the 5th and 95th percentiles, and applied these weights in logistic regression models for each severe outcome using a single variable in indicate the presence of any comorbidity. We used robust standard errors to calculate adjusted odds ratios with 95% confidence intervals and p-values.

Finally, we conducted two additional secondary analyses where we compared the odds of severe outcomes among SARS-CoV-2 patients first to those of influenza A patients only, and second, to those of influenza A/H1 patients only.

Statistical analyses were performed in RStudio using R (version 4.5.1) with the ‘tidyverse’, ‘stats’, ‘nnet’, ‘gtsummary’, and ‘broom’ packages.^23^

### Ethics approval

The study was considered to be enhanced public health surveillance and therefore exempted by the local ethics review committee in Albania, Georgia, Kyrgyzstan, North Macedonia and Kosovo^17^ and was exempted by the WHO Ethical Review Committee (ERC).^3^

### Role of funding source

Staff from the World Health Organization Regional Office for Europe contributed to study design, data management, data analysis, interpretation of the data, writing the manuscript, and the decision to submit the paper for publication. Staff from the US Centers for Disease Control and Prevention were not involved in this analysis or writing of the manuscript.

## Results

### Descriptive analysis

**Fig. 2:**
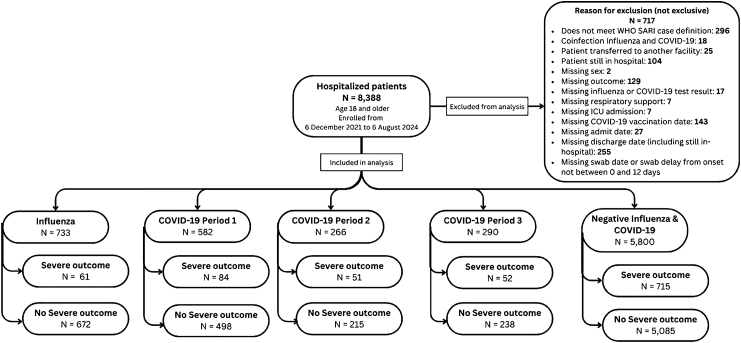
Flow chart of severe acute respiratory infection (SARI) patients included in EuroSAVE COVID-19 vaccine effectiveness analysis, January 2022–November 2023.

From 6 December 2021 to 6 August 2024, 8388 SARI patients were admitted to the surveillance hospitals. After applying our exclusion criteria, a total of 717 patients were excluded (Fig. 2).

Of the 7671 patients included in the analysis, the median age was 64 years (IQR 48–73), 3996 (52%) were male, and 4623 (60%) had at least one comorbidity (Table 1). Most patients were from Albania [4348 (57%)], followed by Georgia [1061 (14%)], Kyrgyzstan [1252 (16%)], Kosovo^17^ [605 (8%)], and North Macedonia [405 (5%)]. Of all patients, 892 (12%) had received a SARS-CoV-2 vaccine in the previous 12 months, and 230 (3%) of the 7360 patients with known influenza vaccination history had received an influenza vaccine in the previous year. COVID-19 vaccination in the previous 12 months was highest during Period 1 [205 (35%)], followed by Period 2 [22 (8%)], and Period 3 [4 (1%)].

**Table 1:** Descriptive characteristics of SARI patients and in-hospital outcomes, EuroSAVE, 2021–2024.

Variable	Overall^a^	Influenza^a^	COVID-19 P1^a^	COVID-19 P2^a^	COVID-19 P3^a^	/COVID-19 & Influenza Negative^a^	p-value^b^
Total	7671	733 (10%)	582 (8%)	266 (3%)	290 (4%)	5800 (76%)	
Age (years)	64 (48, 73)	54 (36, 68)	70 (59, 78)	69 (55, 78)	69 (57, 76)	63 (48, 73)	**<0.001**
Age category (years)							**<0.001**
18–59	3196	425 (13%)	154 (5%)	85 (3%)	84 (3%)	2448 (77%)	
60–69	1883	151 (8%)	138 (7%)	50 (3%)	74 (4%)	1470 (78%)	
70–79	1675	105 (6%)	180 (11%)	82 (5%)	78 (5%)	1230 (73%)	
80 and over	917	52 (6%)	110 (12%)	49 (5%)	54 (6%)	652 (71%)	
Country							**<0.001**
Albania	4348	444 (10%)	395 (9%)	138 (3%)	185 (4%)	3186 (73%)	
Georgia	1061	58 (5%)	0 (0%)	48 (5%)	21 (2%)	934 (88%)	
Kyrgyzstan	1252	95 (8%)	42 (3%)	21 (2%)	12 (1%)	1082 (86%)	
North Macedonia	405	61 (15%)	23 (6%)	4 (1%)	20 (5%)	297 (73%)	
Kosovo^17^	605	75 (12%)	122 (20%)	55 (9%)	52 (9%)	301 (50%)	
Male	3996	372 (9%)	328 (8%)	129 (3%)	144 (4%)	3023 (76%)	0.14
Any comorbidity	4623	391 (8%)	381 (8%)	156 (3%)	220 (5%)	3475 (75%)	**<0.001**
Cancer	121	2 (2%)	17 (14%)	7 (6%)	11 (9%)	84 (69%)	**<0.001**
Diabetes	1182	88 (7%)	119 (10%)	42 (4%)	103 (9%)	830 (70%)	**<0.001**
Heart disease	2413	187 (8%)	155 (6%)	77 (3%)	125 (5%)	1869 (77%)	**<0.001**
Immunodeficiency	45	2 (4%)	1 (2%)	0 (0%)	4 (9%)	38 (84%)	0.11
Liver disease	172	12 (7%)	10 (6%)	4 (2%)	6 (3%)	140 (81%)	0.5
Lung disease (not asthma)	1018	91 (9%)	64 (6%)	21 (2%)	22 (2%)	820 (81%)	**<0.001**
Lung disease (asthma)	975	78 (8%)	39 (4%)	20 (2%)	19 (2%)	819 (84%)	**<0.001**
Neurological condition	378	20 (5%)	60 (16%)	13 (3%)	30 (8%)	255 (67%)	**<0.001**
Renal disease	339	19 (6%)	37 (11%)	11 (3%)	11 (3%)	261 (77%)	**0.02**
Rheumatological condition	1478	154 (10%)	215 (15%)	64 (4%)	129 (9%)	916 (62%)	**<0.001**
Influenza vaccination for current season	230	16 (7%)	10 (4%)	3 (1%)	6 (3%)	195 (85%)	**0.03**
COVID-19 Vaccination within 365 days	892	54 (6%)	205 (23%)	22 (2%)	4 (0%)	607 (68%)	**<0.001**
COVID-19 Vaccination within 365 days	263	19 (7%)	47 (18%)	3 (1%)	0 (0%)	194 (74%)	**<0.001**
Admitted during high admissions month	3905	570 (15%)	473 (12%)	173 (4%)	48 (1%)	2641 (68%)	**<0.001**
Admitted during influenza season	5272	727 (14%)	98 (2%)	243 (5%)	140 (3%)	4064 (77%)	**<0.001**
Severe outcome (Died, ICU, Vent, ECMO)	963	61 (6%)	84 (9%)	51 (5%)	52 (5%)	715 (74%)	**<0.001**
Died in hospital	285	23 (8%)	73 (26%)	12 (4%)	36 (13%)	141 (49%)	**<0.001**
Admitted to ICU	749	42 (6%)	20 (3%)	43 (6%)	39 (5%)	605 (81%)	**<0.001**
Required mechanical ventilation or ECMO	202	18 (9%)	15 (7%)	5 (2%)	27 (13%)	137 (68%)	**<0.001**

Not included in statistical models due to low vaccination coverage among study participants and 82 participants with missing values for vaccine coverage.

a Continuous variable: median (interquartile range); categorical variables: number of patients or number of patients (percentage of patients in category by PCR test result group).

b Chi-square analysis p-value; bold text indicates value is <0.05.

Of all 7671 patients, 1138 (15%) tested positive for SARS-CoV-2, 741 (10%) tested positive for influenza, and 5800 (76%) were negative for both viruses. Of the 733 influenza-positive patients, 593 (83%) were influenza A and 120 (2%) were influenza B, and the type was unrecorded for 20 cases. Of the 338 (46%) influenza A specimens where subtyping information was available, 71 (21%) were A/H3 and 267 (79%) were A/H1. Overall, 963 (13%) patients had a severe outcome; 749 (10%) patients were admitted to the ICU, 202 (3%) received mechanical ventilation or ECMO, and 285 (4%) died (Table 1).

Omicron subvariants predominated in all three COVID-19 waves. According to sequencing data available from the 5 countries and areas retrieved from the Global Initiative on Sharing All Influenza Data (GISAID) EpiCoV™ database,^24^ Period 1 was characterized by the circulation of the Delta (B.1.617.2) in early December 2021 followed by the predominance of the first SARS-CoV-2 Omicron variants, BA.1, BA.2, and BA.5. Period 2 coincided with the rise in dominance of Omicron XBB subvariants (especially XBB.1.5) and other Omicron variants. During Period 3, a mix of new, briefly circulating Omicron variants (Omicron FL, and Omicron JN) predominated (Fig. 1).

Among the five test result groups analyzed, either by wave (COVID-19 Period 1, COVID-19 Period 2, COVID-19 Period 3) or test positivity (influenza-positive, and SARS-CoV-2- and influenza-negative patients), the median age was highest in COVID-19 Period 1 [70 years (IQR 59,78)]. The percentage of males was relatively consistent across all five groups [44–51%, (p-value = 0.2)], and the percentage of patients with at least one comorbidity ranged from 54% (Influenza) to 73% (COVID-19 Period 3) (p-value ≤ 0.001) (Table 2).

**Table 2:** Crude ad adjusted odds ratios for in-hospital severe outcome, EuroSAVE, 2021–2024.

Variable	Severe outcome			Univariable analysis		Multivariable analysis	
	Overall^a^	No^a^	Yes^a^	OR^b^ (95% CI)	p-value^c^	aOR^b^ (95% CI)	p-value^c^
PCR test result							
Influenza	733	672 (92%)	61 (8%)	ref		ref	
COVID-19 P1	582	498 (86%)	84 (14%)	1.86 (1.31–2.64)	**<0.001**	1.80 (1.23–2.64)	**<0.01**
COVID-19 P2	266	215 (81%)	51 (19%)	2.61 (1.74–3.91)	**<0.001**	1.94 (1.25–3.01)	**<0.01**
COVID-19 P3	290	238 (82%)	52 (18%)	2.41 (1.61–3.58)	**<0.001**	1.63 (1.05–2.54)	**0.03**
COVID-19 & Influenza Negative	5800	5085 (88%)	715 (12%)	1.55 (1.19–2.06)	**<0.01**	1.10 (0.83–1.49)	0.51
Age category (years)							
18–59	3196	2952 (92%)	244 (8%)	ref		ref	
60–69	1883	1646 (87%)	237 (13%)	1.74 (1.44–2.10)	**<0.001**	1.43 (1.16–1.76)	**<0.001**
70–79	1675	1402 (84%)	273 (16%)	2.36 (1.96–2.83)	**<0.001**	1.86 (1.51–2.29)	**<0.001**
80 and over	917	708 (77%)	209 (23%)	3.57 (2.92–4.37)	**<0.001**	2.57 (2.04–3.23)	**<0.001**
Male	3996	3446 (86%)	550 (14%)	1.26 (1.10–1.45)	**<0.001**	1.30 (1.12–1.50)	**<0.001**
Received COVID-19 vaccination (365 days)	892	819 (92%)	73 (8%)	0.59 (0.46–0.75)	**<0.001**	0.67 (0.51–0.88)	**<0.01**
High admissions month	3905	3446 (88%)	459 (12%)	0.86 (0.75–0.99)	**0.03**	1.00 (0.86–1.17)	0.97
Cancer	121	88 (73%)	33 (27%)	2.67 (1.76–3.96)	**<0.001**	2.01 (1.27–3.10)	**<0.01**
Diabetes	1182	932 (79%)	250 (21%)	2.17 (1.85–2.55)	**<0.001**	1.78 (1.48–2.13)	**<0.001**
Heart disease	2413	1957 (81%)	456 (19%)	2.18 (1.90–2.50)	**<0.001**	1.63 (1.38–1.92)	**<0.001**
Immunodeficiency	45	31 (69%)	14 (31%)	3.18 (1.63–5.88)	**<0.001**	3.73 (1.77–7.43)	**<0.001**
Liver disease	172	136 (79%)	36 (21%)	1.88 (1.27–2.70)	**<0.001**	1.44 (0.95–2.12)	0.07
Lung disease (not asthma)	1018	805 (79%)	213 (21%)	2.08 (1.76–2.46)	**<0.001**	2.31 (1.63–3.24)	**<0.001**
Lung disease (Asthma)	975	792 (81%)	183 (19%)	1.75 (1.46–2.09)	**<0.001**	0.41 (0.28–0.60)	**<0.001**
Neurological condition	378	263 (70%)	115 (30%)	3.32 (2.63–4.17)	**<0.001**	3.08 (2.38–3.97)	**<0.001**
Renal disease	339	264 (78%)	75 (22%)	2.06 (1.57–2.68)	**<0.001**	1.46 (1.09–1.93)	**<0.01**
Rheumatological condition	1478	1306 (88%)	172 (12%)	0.90 (0.75–1.07)	0.24	1.29 (1.04–1.60)	**0.02**

The country variable is included in the model but the results are not shown.

a Number of participants (Percent of total participants).

b Odds ratio (OR) or adjusted odds ratio (aOR) with 95% confidence intervals (CI); the reference group for binary variables (e.g., sex, vaccination status, comorbidities) consists of patients with a negative response to the variable (e.g., no diabetes for the diabetes variable).

c p-values based on Wald tests; bold text indicates value is <0.05.

### Comparison of in-hospital outcomes

The percentage of patients with a severe outcome ranged from a high of 19% in COVID-19 Period 2 patients to a low of 8% in influenza patients (chi-square p-value < 0.001). In-hospital mortality was highest in COVID-19 Period 1 (13%) patients and lowest in the influenza (4%) and SARS-CoV-2- and influenza-negative patients (2%) (chi square p-value < 0.001) (Table 1).

Compared to influenza patients, COVID-19 patients were more likely to have severe outcomes in Period 1 [aOR = 1.80 (95% CI 1.23–2.64)], Period 2 [aOR = 1.94 (95% CI 1.25–3.01)], and Period 3 [aOR = 1.63 (95% CI 1.05–2.54)] (Table 2). The risk of severe outcomes was not different for SARS-CoV-2- and influenza-negative patients compared to influenza patients [aOR = 1.10 (95% CI 0.83–1.49)].

COVID-19-patients in Period 1 [aOR = 2.80 (95% CI 1.67–4.82)] and Period 3 [aOR = 2.25 (95% CI 1.23–4.18)] were more likely to die in hospital compared to influenza patients (Table 3). The aORs for Period 2 COVID-19 [aOR = 1.04 (95% CI 0.48–2.15)] and SARS-CoV-2- and influenza-negative patients [aOR = 0.74 (95% CI 0.47–1.21)] were not significantly different compared to influenza patients.

**Table 3:** Crude and adjusted odds ratios for in-hospital death, EuroSAVE, 2021–2024.

Variable	Died			Univariable analysis		Multivariable analysis	
	Overall^a^	No^a^	Yes^a^	OR^b^ (95% CI)	p-value^c^	aOR^b^ (95% CI)	p-value^c^
PCR test result							
Influenza	733	710 (97%)	23 (3%)	ref		ref	
COVID-19 P1	582	509 (87%)	73 (13%)	4.43 (2.78–7.32)	**<0.001**	2.80 (1.67–4.82)	**<0.001**
COVID-19 P2	266	254 (95%)	12 (5%)	1.46 (0.69–2.92)	0.30	1.04 (0.48–2.15)	0.93
COVID-19 P3	290	254 (88%)	36 (12%)	4.38 (2.56–7.62)	**<0.001**	2.25 (1.23–4.18)	**<0.01**
COVID-19 & influenza negative	5800	5659 (98%)	141 (2%)	0.77 (0.50–1.23)	0.25	0.74 (0.47–1.21)	0.20
Age category (years)							
18–59	3196	3141 (98%)	55 (2%)	ref		ref	
60–69	1883	1812 (96%)	71 (4%)	2.24 (1.57–3.21)	**<0.001**	1.41 (0.96–2.09)	0.08
70–79	1675	1591 (95%)	84 (5%)	3.02 (2.14–4.28)	**<0.001**	1.59 (1.08–2.34)	**0.02**
80 and over	917	842 (92%)	75 (8.2%)	5.09 (3.57–7.29)	**<0.001**	2.71 (1.82–4.05)	**<0.001**
Male	3996	3821 (96%)	175 (4%)	1.48 (1.17–1.90)	**<0.01**	1.44 (1.12–1.87)	**<0.01**
Received COVID-19 Vaccination (365 days)	892	862 (97%)	30 (3%)	0.89 (0.59–1.29)	0.55	0.63 (0.40–0.97)	**0.04**
High admissions month	3905	3740 (96%)	165 (4%)	1.34 (1.06–1.71)	0.20	1.23 (0.93–1.64)	0.14
Cancer	121	111 (92%)	10 (8%)	2.38 (1.16–4.38)	**0.01**	1.66 (0.77–3.24)	0.17
Diabetes	1182	1070 (91%)	112 (9%)	3.82 (2.98–4.88)	**<0.001**	2.05 (1.53–2.72)	**<0.001**
Heart disease	2413	2264 (94%)	149 (6%)	2.48 (1.95–3.15)	**<0.001**	1.53 (1.14–2.06)	**<0.01**
Immunodeficiency	45	38 (84%)	7 (16%)	4.87 (1.98–10.3)	**<0.001**	4.35 (1.54–10.8)	**<0.01**
Liver disease	172	159 (92%)	13 (8%)	2.17 (1.16–3.73)	**<0.01**	2.81 (1.38–5.27)	**<0.01**
Lung disease (not asthma)	1018	974 (96%)	44 (4%)	1.20 (0.86–1.65)	0.27	1.73 (1.01–2.86)	**0.04**
Lung disease (Asthma)	975	946 (97%)	29 (3%)	0.77 (0.51–1.12)	0.19	0.42 (0.22–0.79)	**<0.01**
Neurological condition	378	330 (87%)	48 (13%)	4.33 (3.08–5.97)	**<0.001**	2.76 (1.90–3.96)	**<0.001**
Renal disease	339	300 (88%)	39 (12%)	3.74 (2.59–5.29)	**<0.001**	2.84 (1.88–4.19)	**<0.001**
Rheumatological condition	1478	1368 (93%)	110 (7%)	2.77 (2.16–3.53)	**<0.001**	2.16 (1.58–2.95)	**<0.001**

The country variable is included in the model but the results are not shown.

a Number of participants (Percent of total participants).

b Odds ratio (OR) or adjusted odds ratio (aOR) with 95% confidence intervals (CI); the reference group for binary variables (e.g., sex, vaccination status, comorbidities) consists of patients with a negative response to the variable (e.g., no diabetes for the diabetes variable).

c p-values based on Wald tests; bold text indicates value is <0.05.

SARI patients aged ≥60 years were more likely to have severe outcomes compared to SARI patients aged 18–59 years, with aORs of 1.43 (95% CI 1.16–1.76) for patients aged 60–69 years, 1.86 (95% CI 1.51–2.29) for patients 70–79 years old, and 2.57 (95% CI 2.04–3.23) for patients ≥80 years old. Males were also more likely to have severe outcomes than females [aOR = 1.30 (95% CI 1.12–1.50). Among SARI patients with comorbidities, those with immunodeficiency, neurological conditions and lung disease (excluding asthma) were most likely to have severe outcomes. In contrast, having received at least one COVID-19 vaccine in the previous 12 months was associated with a reduced odds of severe outcome [aOR = 0.66 (95% CI 0.50–0.86)] (Table 2). Compared with SARI patients aged 18–59 years, the odds of death was higher among older patients. Like in the analysis of severe outcomes, males had a higher likelihood of death compared to females, patients with certain chronic conditions had statistically significantly higher odds of in-hospital death, and patients who had received a COVID-19 vaccine within 365 days had lower odds of in-hospital death (Table 3).

### Secondary and sensitivity analyses

In our analysis of influenza patients by year, influenza patients had a relatively similar percentage of severe outcomes across years. Among 390 influenza-positive SARI patients in the 2022–2023 season, 29 (7%) had a severe outcome and 11 (3%) died (Supplementary Tables S1 and S2). Among 328 patients in the 2023–2024 season 32 (10%) had a severe outcome and 12 (4%) died. There was no significant difference in the risk of severe outcome [aOR = 0.89 (95% CI 0.46–1.71)] or death [aOR = 0.63 (95% CI 0.24–1.65)] for patients admitted in the 2023–2024 season compared to those admitted during the 2022–2023 season. As there were only 15 influenza-positive SARI patients in the 2021–2022 season, we omitted this season from the analysis.

In our secondary analysis that focused on individuals recommended for influenza vaccine, we found that, similar to our main analysis, the odds of severe outcome were elevated among patients with COVID-19 in Period 1 and Period 2 compared to patients with influenza; however in this analysis the difference was not statistically significant (p-value < 0.05) in Period 3 (Supplementary Table S3). For death, the results were similar to those of our main analysis (Supplementary Table S4). In our stratified analysis by age, we found that among patients ≥60 years old, results were similar to those in the primary analysis, except that COVID-19 Period 3 patients did not have a statistically significant higher risk of severe outcome compared to influenza patients (Supplementary Tables S5 and S6). Among patients 18–59 years old, the sample size was relatively small, and 95% CIs were wide, and therefore the results did not provide evidence to support a difference in outcomes between any of the PCR test-result groups for severe outcome or death among this subpopulation (Supplementary Tables S7 and S8).

In our sensitivity analysis that defined COVID-19-vaccinated patients as those who had received a vaccine within 6 months prior to their SARI admission (Supplementary Tables S9 and S10), and our analysis in which we included age as a continuous variable (Supplementary Tables S11 and S12), results were similar to those of the main analysis.

Likewise, in our sensitivity analysis where we accounted for the nested structure of the data for hospitals within countries (Supplementary Tables S13 and S14), our sensitivity analysis where we removed SARI patients who had been recently vaccinated against COVID-19 and influenza (Supplementary Tables S15 and S16), and our sensitivity analysis where we adjusted for differences between PCR test-result groups using inverse probability weighting (IPW) (Supplementary Tables S17 and S18), our results were similar to the main analysis for both severe outcome and death, with the exception of wide 95% CIs in our aOR for COVID-19 Period 2 relative to influenza in the analysis of severe outcome.

## Discussion

We found that in five middle-income countries and areas in the eastern part of the WHO European Region, from 2021 to 2024, a period characterized by the circulation of Omicron variants and subvariants, hospitalized COVID-19 patients were more likely to have severe outcomes compared to influenza-positive patients. Furthermore, in two of the three periods we evaluated (Period 1 and Period 3), COVID-19 patients were more likely to die in-hospital compared to influenza patients. These findings demonstrate the continued severity of COVID-19 in hospitalized patients and underscore the importance of promoting interventions that continue to be shown to reduce poor outcomes from COVID-19, such as annual up-to-date COVID-19 vaccine,^25^, ^26^, ^27^, ^28^, ^29^ and early antiviral treatment.^30^

Our findings align with similar studies that have consistently found that from the beginning of the pandemic, COVID-19 resulted in more severe outcomes compared to influenza, although the relative risk of severe disease for COVID-19 has decreased over time.^7^^,^^8^^,^^10^^,^^31^^,^^32^ A study conducted during 2021–2024 in Denmark found that even as recently as the 2023–2024 winter season, patients hospitalized with COVID-19 had a higher relative 30-day mortality compared to influenza patients (adjusted incidence rate ratio 1.58 (95% CI 1.28–1.95)).^9^ Unlike our study, the Danish study included all hospitalized patients rather than only SARI patients. A study from the US, which, unlike our study, included mostly males (93%), found that from October 2023 to March 2024, hospitalized COVID-19 patients had a higher risk of death compared with hospitalized influenza patients (adjusted death rate 5.70% vs 4.24% at 30 days; adjusted Hazard Ratio 1.35 (95% CI 1.10–1.66)).^10^ While some of the above studies differ slightly from our study in methodology and time periods, these studies have consistently found that COVID-19 continues to be associated with severe outcomes compared to influenza, even in the post-pandemic period.

In our analysis, the aOR for COVID-19 relative to influenza in Period 2 was lower than in Period 1 and Period 3. This lower aOR may be an artifact of the smaller sample size and few reported deaths in Period 2, which also produced wide 95% CIs. These wide 95% CIs overlap with the 95% CIs for the Period 1 and Period 3 results, making it difficult to conclude whether the lower aOR for Period 2 reflects a true difference.

In our sensitivity analyses where we restricted our analyses to patients recommended for vaccination and patients ≥60 years, we found a higher risk for severe outcome among hospitalised SARS-CoV-2-positive patients compared to influenza-positive patients in Periods 1 and 2 but not in Period 3. In both sensitivity analyses, in Period 1 and Period 3, the risk of death was higher for COVID-19 patients compared to influenza patients. These findings underscore the risk of severe COVID-19 in these two sub-populations that are targeted for COVID-19 vaccination.

In analyses restricted to influenza type A and to subtype A/H1, COVID-19-positive SARI patients had a higher risk of severe outcome and death, consistent with the main analysis. Although the direction of effect was similar, the confidence intervals were wide in the A/H1 analysis, likely because of the smaller sample size; there were only 267 influenza A/H1-positive SARI patients. Because of the small sample size and potential selection bias from incomplete subtyping results, the results of this analysis should be interpreted with caution.

SARI patients in our study had very low rates of COVID-19 vaccine uptake; only 12% of SARI patients had received a COVID-19 vaccine in the past 12 months, and in the last period of our study (Period 3), only 1.4% of patients had received a COVID-19 vaccine in the past 12 months. These low vaccination rates reflect missed opportunities to prevent severe illnesses, as COVID-19 continues to cause severe disease, even more so than influenza in this analysis. Up-to-date COVID-19 vaccination was shown to be 70.7% (95% CI 66.6–74.3) effective in preventing severe disease in high-risk individuals in a recent analysis in the United States (US).^33^ Additionally, a recent analysis with EuroSAVE network data reported a 60.0% VE (95% CI 32.2–76.4) for patients who received their last COVID-19 vaccine 90–179 days prior to hospitalization. In this study VE waned when the most recent vaccine dose was more than 179 days prior to hospitalization, which may indicate the need for careful timing of vaccine doses or a more frequent vaccine schedule for those with risk factors.^3^

While the low vaccine uptake in patients in our study, which included mostly adults ≥60 years old and adults with comorbidities -- populations at high risk for poor outcomes from SARS-CoV-2 infection -- may reflect vaccine hesitancy and a lack of appreciation of the potential of COVID-19 to cause severe disease, other factors may be involved. A recent analysis using data from the EuroSAVE network found that, as of October 2024, out of six countries and areas, four had not updated their national COVID-19 vaccination policies to recommend annual vaccine for high-risk individuals, according to WHO Strategic Advisory Group of Experts (SAGE) on Immunization guidelines.^34^ In addition, in four of the six countries, COVID-19 vaccines were simply not available. Efforts should be made to better understand the main barriers to COVID-19 vaccination, and to ensure vaccine availability and update vaccine recommendations in order to increase up-to-date vaccination in high-risk groups.

We found that older age, male sex, and certain chronic conditions increased the risk of severe outcomes, including death, among hospitalized SARI patients, findings that have been described in previous studies. A study conducted in the Kingdom of Bahrain from 2018 to 2021 found that SARI patients who were ≥50 years old, and SARI patients with chronic heart disease, hematological disorders, lung diseases, and kidney diseases had an increased risk of mechanical ventilation, ICU admission, or death.^31^ A study of SARI patients hospitalized in five countries in the WHO Eastern Mediterranean region during 2007–2014 found that patients ≥50 years old and those with self-reported pre-existing chronic diseases were more likely to experience severe outcomes, such as requiring mechanical ventilation, ICU admission, and/or death.^35^ Our findings from middle-income countries in the WHO European Region reinforce these results from other regions in the world.

Our study has a number of strengths. We used standardized case definitions, recruitment protocols, testing algorithms, and data collection methods across five countries and areas. Patients were recruited in established routine respiratory virus surveillance hospitals through the EuroSAVE network, using nearly identical questionnaires, which ensured standardization among sites in the five countries and areas. Second, we included data from a broad range of middle-income countries across the eastern part of the WHO European Region, an area of the world where little evidence is available about in-hospital severe outcomes from COVID-19 and influenza. Finally, pooling results across multiple countries and areas improved the power of the study and enabled a more robust analysis compared to a single-country analysis.

Our study also has some limitations. The hospitals included in each of the five countries and area were nearly all in large cities; therefore, our results may not be generalizable to their entire populations. However, a previously published analysis found that national COVID-19 primary series and booster coverage rates in the five countries and areas included in our study were similar to the coverage rates among patients admitted to participating EuroSAVE hospitals in the same five countries and areas^36^ Additionally, we were not able to evaluate differences in in-hospital management protocols for COVID-19 and influenza, in particular in regards to criteria for ICU admission, ECMO use, and use of antiviral medication, which may have varied in different hospitals within the same country and between countries. If these unmeasured differences variably impacted hospital outcome, then in-hospital outcomes in our study may not be representative of all five countries and areas. Furthermore, although we included country of hospitalization in our multivariable model, possible differences in in-hospital management between countries could have influenced the study outcomes in ways that we could not characterize or adjust for. Other unmeasured differences in practices in different countries may partly explain differences in rates of severe disease across periods. Outcomes, including death, were only recorded during the patients’ hospital stay, and we therefore could not account for deaths that occurred following discharge or transfer. Differences in recent COVID-19 vaccine coverage among the three COVID-19 periods may have impacted COVID-19 severity. However, we did adjust for COVID-19 vaccination status in the model.

Additional limitations include the fact that we did not adjust for influenza vaccine, which could impact the severity of disease. However, influenza vaccination coverage among patients included in our study was very low (3%) and therefore unlikely to have significantly impacted overall outcomes. We could not evaluate relative severity of COVID-19 by variant because of small sample size; however, we attempted to group similar variants and subvariants in the same analysis period. Other sources of potential bias include the self-reporting of comorbidities, self-reporting of influenza vaccination status in some sites, and possible changes in health seeking behavior over the course of the study period as perceptions of risk related to respiratory infections changed. Sensitivity of SARS-CoV-2 PCR can be low in the early stages of infection, and therefore we may have incorrectly classified some patients as SARS-CoV-2-negative if they were hospitalized and sampled early after their infection. We were unable to collect information on socioeconomic status or prior SARS-CoV-2 infection. We were not able to conduct additional viral or bacterial testing to better characterize the SARI patients who were negative for SARS-CoV-2 and influenza. This group constituted the vast majority of SARI patients; future similar analyses should attempt to conduct additional pathogen testing in order to better characterise this group. Because of the above limitations, particularly our inability to control for a number of important confounding variables, our findings should be interpreted with caution.

### Conclusion

We found that COVID-19 caused more severe outcomes, including death, compared to influenza and SARS-CoV-2- and influenza-negative patients among SARI patients during the study period, including 2023 and 2024, in five middle-income countries and areas in the WHO European Region. Despite the high relative severity of COVID-19, COVID-19 vaccination rates were low, particularly in 2023 and 2024. Our findings highlight the continued need to optimize preventive and therapeutic measures for COVID-19.

## Contributors

AG and MAK led conceptualisation and methodology. AG and MAK led data curation, formal analysis, and writing—original draft. PM, JEF, JH, AMCR, SC, MAW, OA, IF, and MAK contributed to conceptualisation, methodology, formal analysis, and contributed to writing—review & editing. KM, OTM, BK, KS, DO, NC, KZ, AK, EJ, and AR contributed to investigation and data collection in their respective countries and areas, and contributed to writing—review & editing.

AG, MAK, SC, JH, and AMCR had access to all the data. AG and MAK verified the underlying data. AG wrote all data management and analysis code. MAK provided supervision.

AG and MAK were responsible for the decision to submit the manuscript.

## Data sharing statement

The data analysed in this study are individual-level patient data collected through national surveillance systems in participating countries and areas of the EuroSAVE Network. These data are subject to national data protection laws, ethical approvals, and data sharing agreements that prohibit onward sharing of patient-level data.

## Declaration of generative AI and AI-assisted technologies in the manuscript preparation process

During the preparation of this work the author(s) used ChatGPT 5.2 to proofread drafts for spelling and grammar, and to improve the flow and clarity of the text. After using this tool/service, the author(s) reviewed and edited the content as needed and take(s) full responsibility for the content of the published article.

## Declaration of interests

The authors declare no competing interests.
