# Supplementary material for: Comparing outcomes of COVID-19 and influenza among hospitalized adults in the European severe acute respiratory infection vaccine effectiveness (EuroSAVE) network, 2021–2024: a retrospective cohort analysis
Source: Lancet Reg Health Eur. 2026 Apr 16;65:101672. doi: 10.1016/j.lanepe.2026.101672 (PMC13099496; doi:10.1016/j.lanepe.2026.101672)

## Supplementary tables

#### Supplementary Table 1. Crude and adjusted odds ratios for in-hospital severe outcomes, restricted to patients PCR positive for influenza, by season, with variables indicating current influenza vaccination (excluding patients with missing data) and the presence of any comorbidities included in the main model, EuroSAVE, 2021-2024.


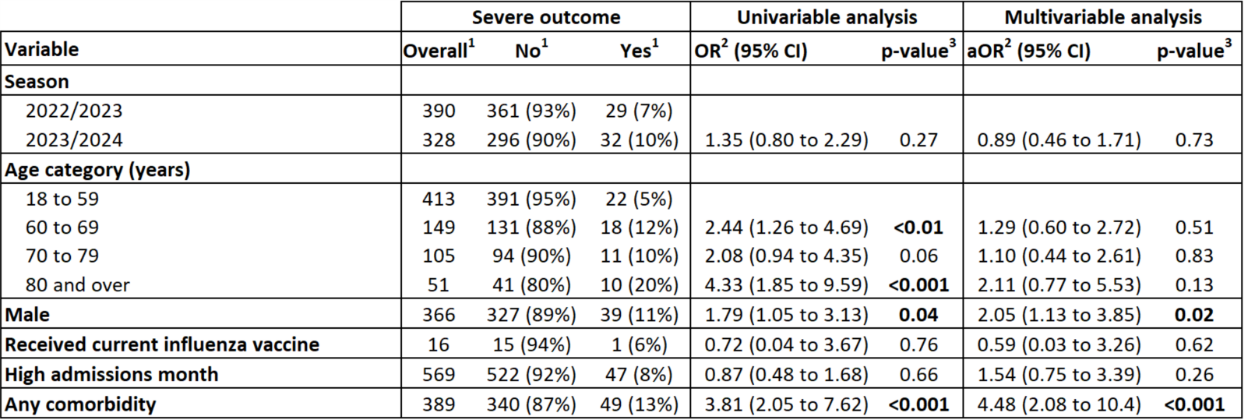


#### Supplementary Table 2. Crude and adjusted odds ratios for in-hospital death, restricted to patients PCR positive for influenza, by season with a variable indicating the presence of any comorbidities included in the main model (a variable indicating recent influenza vaccination was not included due to few deaths), EuroSAVE, 2021-2024.


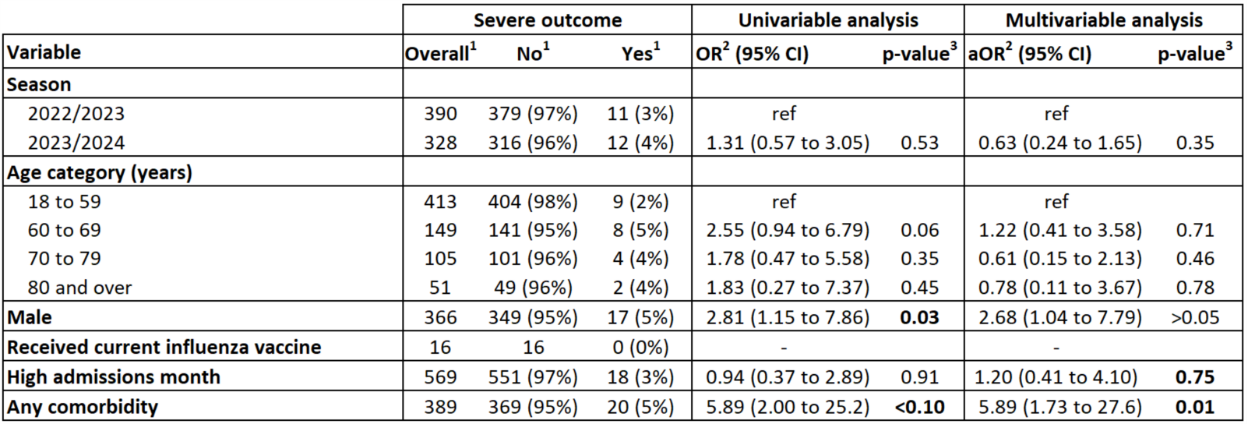


#### Supplementary Table 3. Crude and adjusted odds ratios for in-hospital severe outcomes, restricted to patients age 60 and over or with at least one comorbidity that was included in the primary analysis, EuroSAVE, 2021-2024.


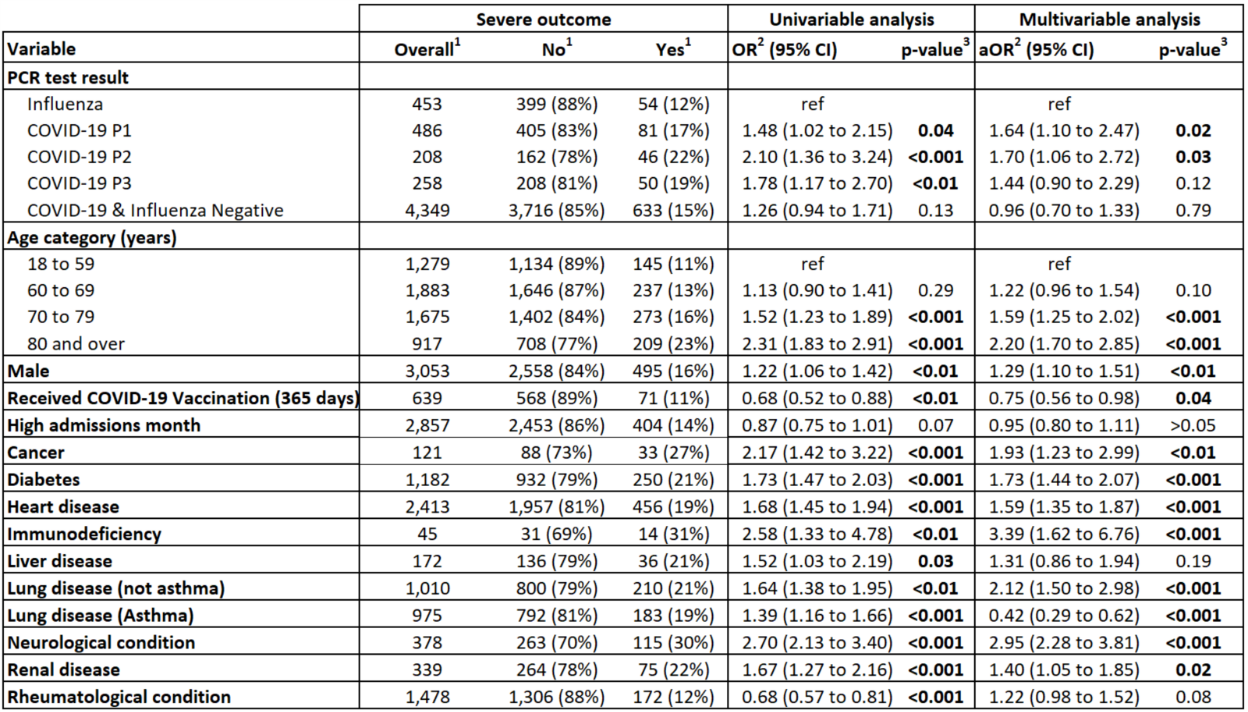


#### Supplementary Table 4. Crude and adjusted odds ratios for in-hospital death, restricted to patients age 60 and over or with at least one comorbidity that was included in the primary analysis, EuroSAVE, 2021-2024.


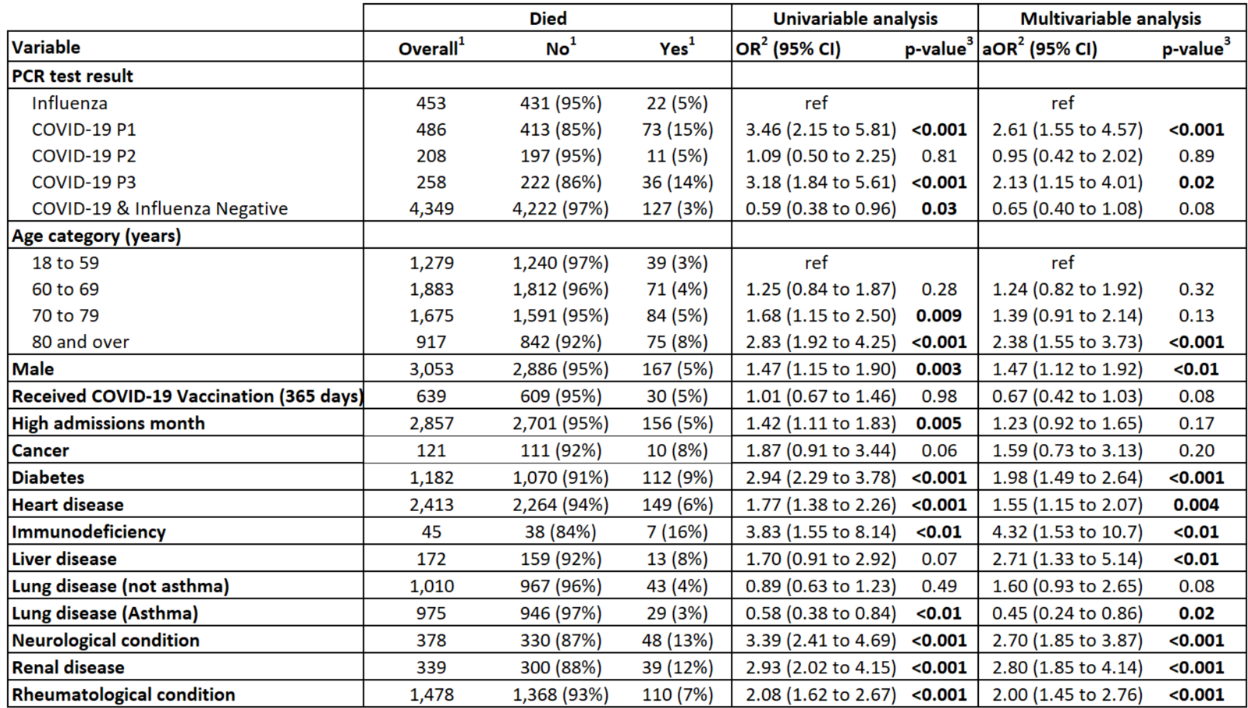


#### Supplementary Table 5. Crude and adjusted odds ratios for in-hospital severe outcomes, restricted to patients age 60 and over with an additional variable for current influenza vaccination, EuroSAVE, 2021-2024.


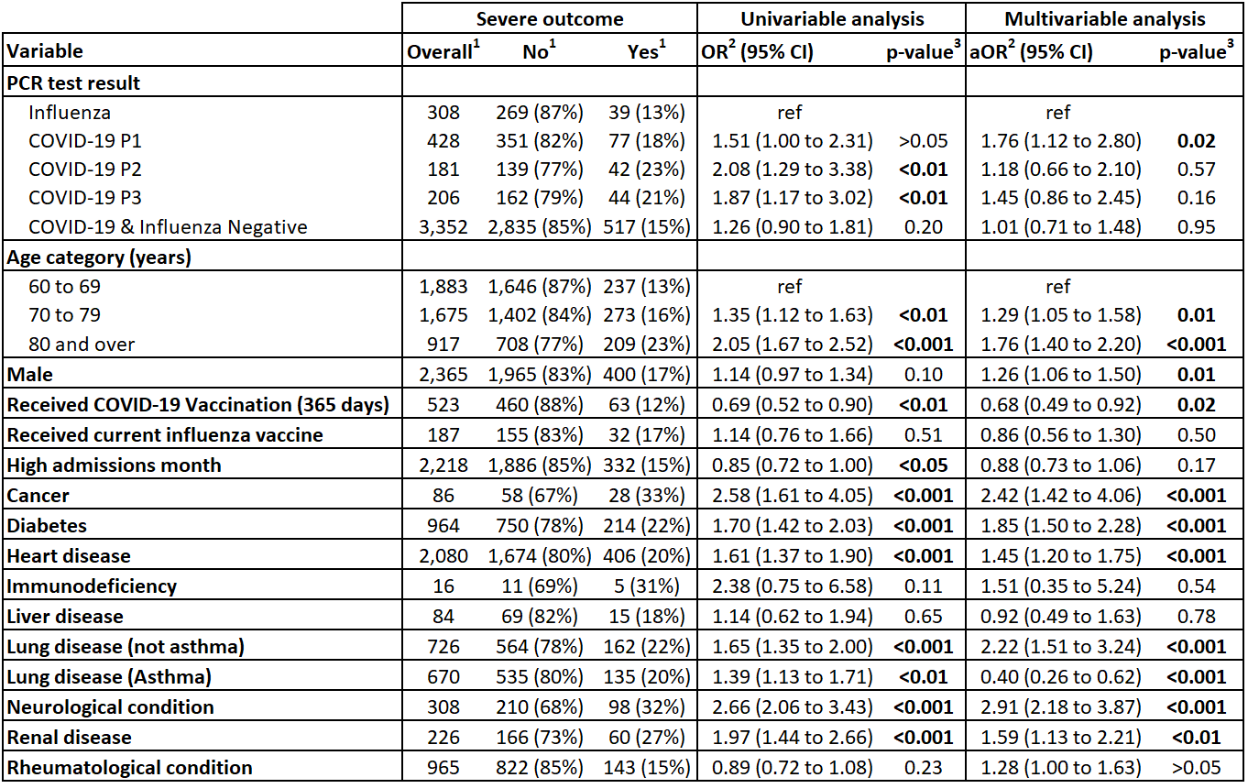


#### Supplementary Table 6. Crude and adjusted odds ratios for in-hospital death, restricted to patients age 60 and over with an additional variable for current influenza vaccination , EuroSAVE, 2021-2024.


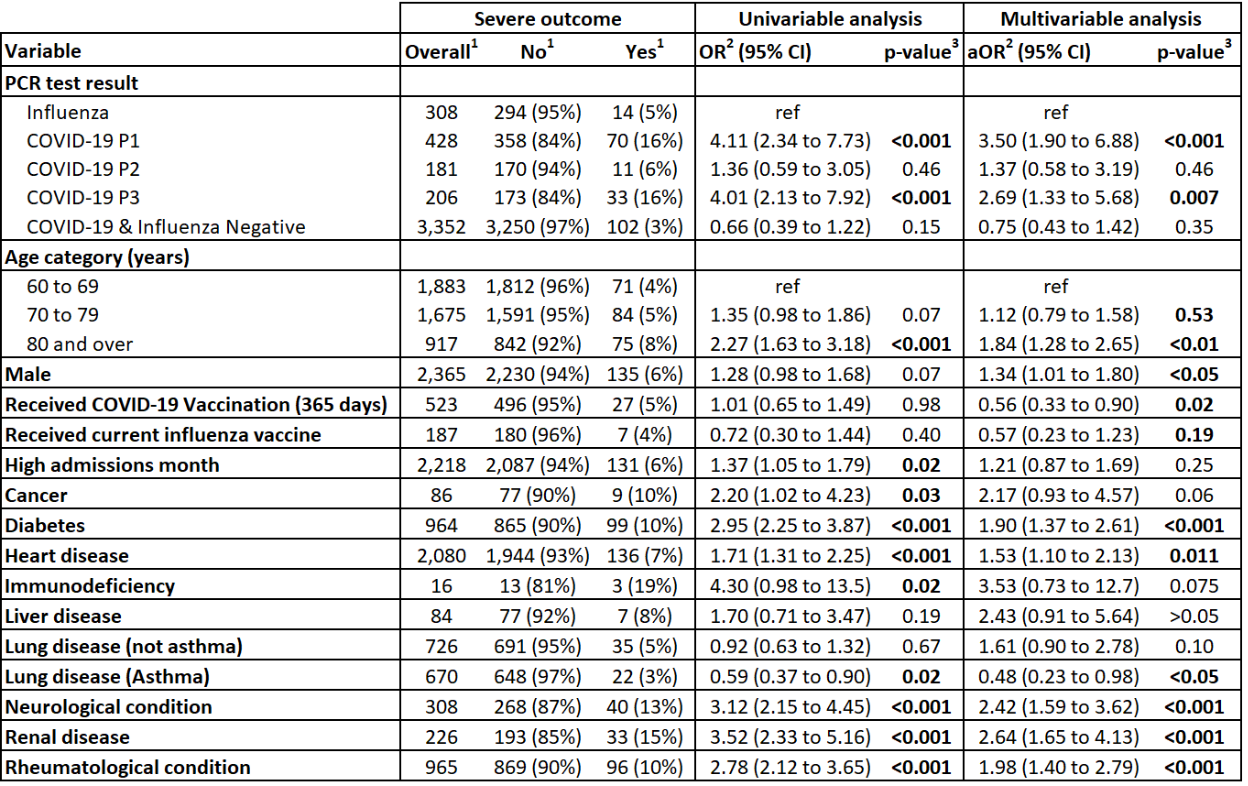


#### Supplementary Table 7. Crude and adjusted odds ratios for in-hospital severe outcomes, restricted to patients age 18 to 59 with an additional variable for current influenza vaccination, EuroSAVE, 2021-2024


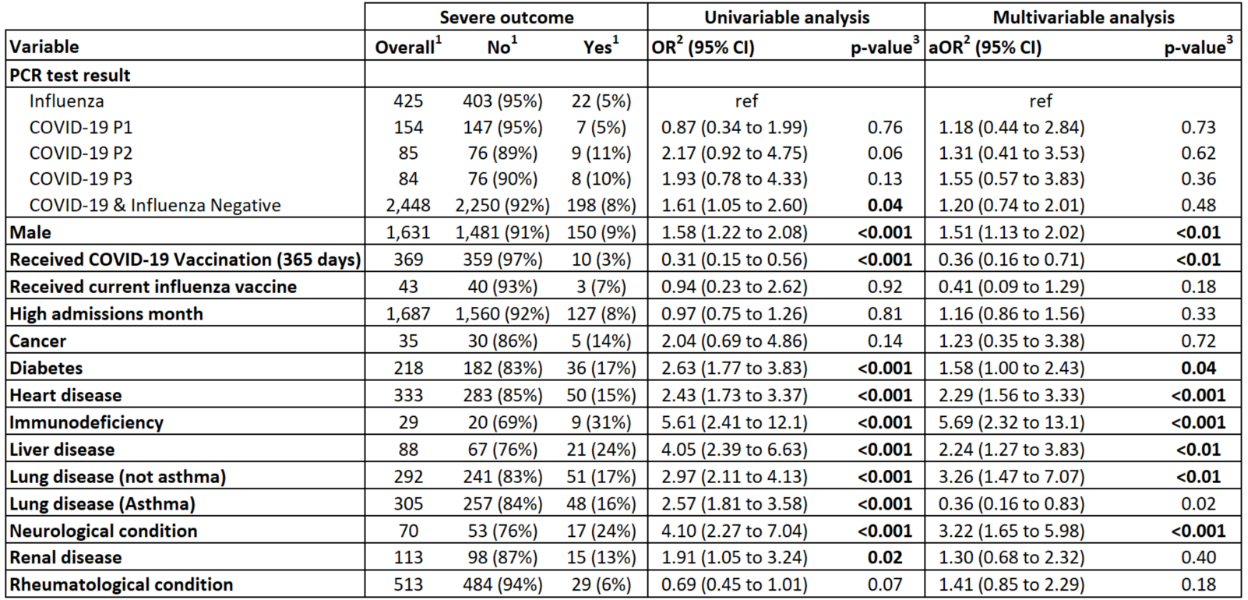


#### Supplementary Table 8. Crude and adjusted odds ratios for in-hospital death, restricted to patients age 18 to 59 with an additional variable for current influenza vaccination, EuroSAVE, 2021-2024.


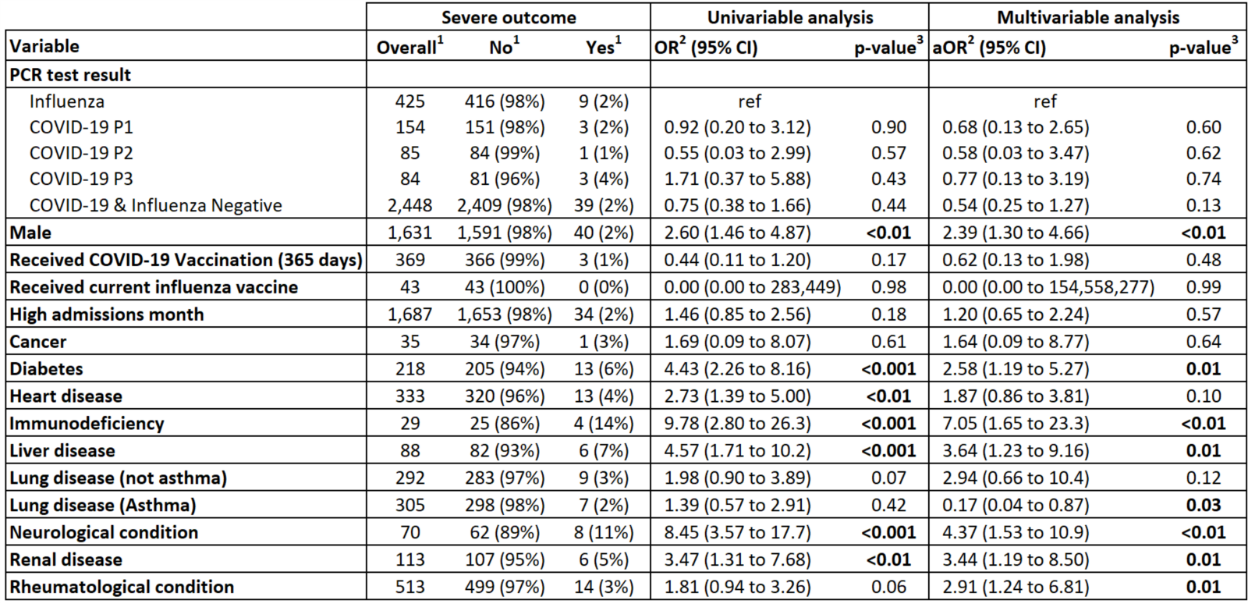


#### Supplementary Table 9. Crude and adjusted odds ratios for in-hospital severe outcomes with a variable indicating COVID-19 vaccination received within 180 days of symptom onset, EuroSAVE, 2021-2024.


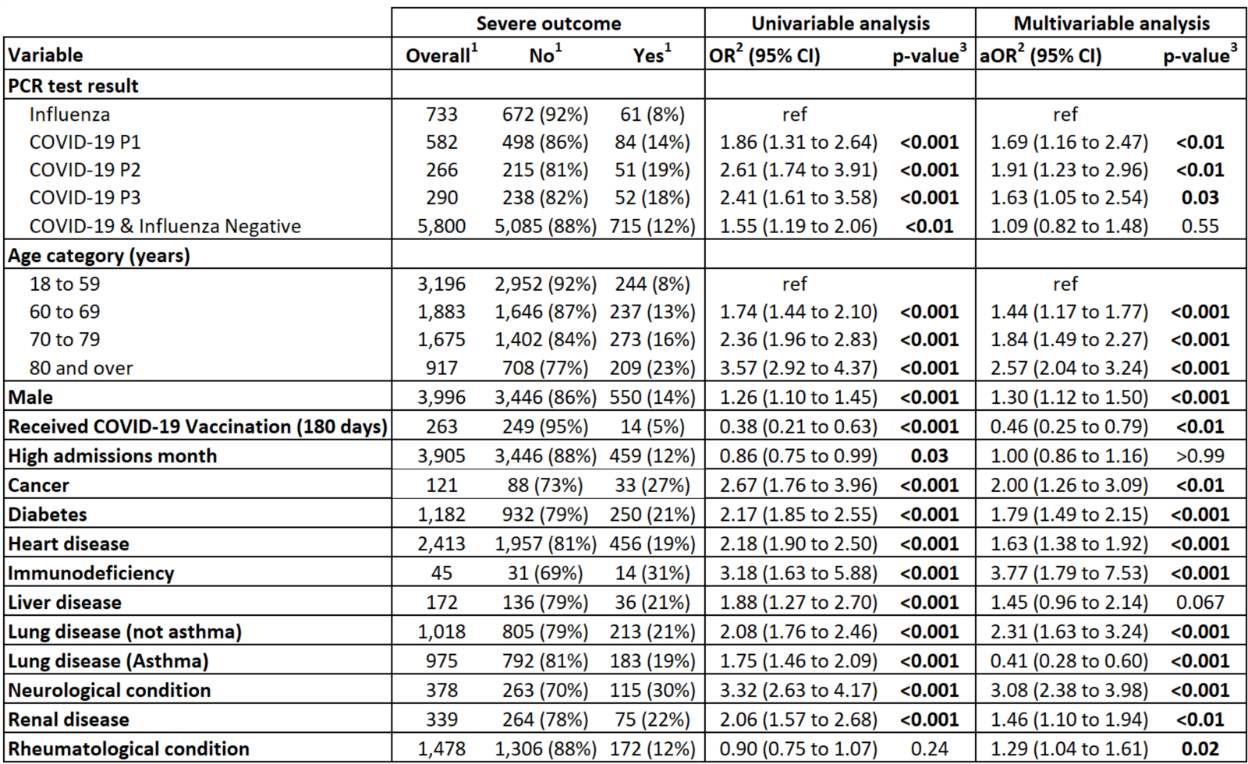


#### Supplementary Table 10. Crude and adjusted odds ratios for in-hospital death with a variable indicating COVID-19 vaccination received within 180 days of symptom onset, EuroSAVE, 2021-2024.


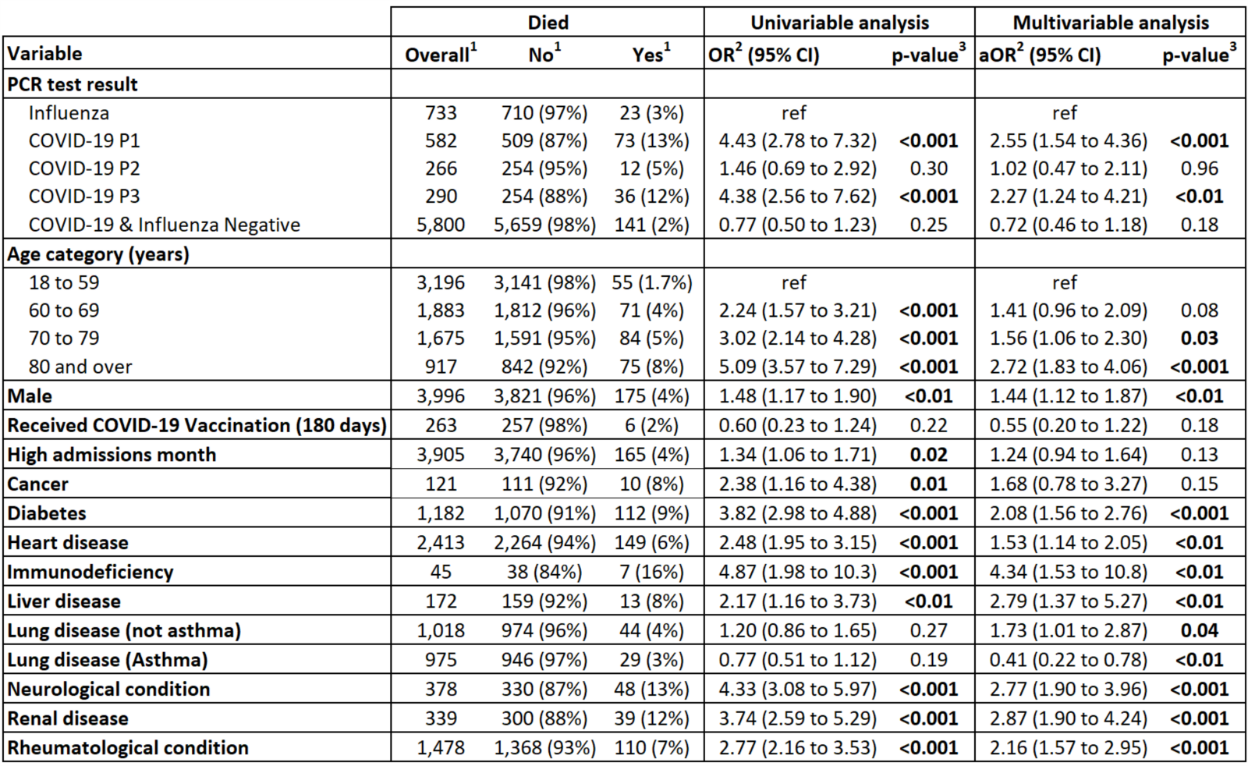


#### Supplementary Table 11. Crude and adjusted odds ratios for in-hospital severe outcomes with a continuous age variable, EuroSAVE, 2021-2024.


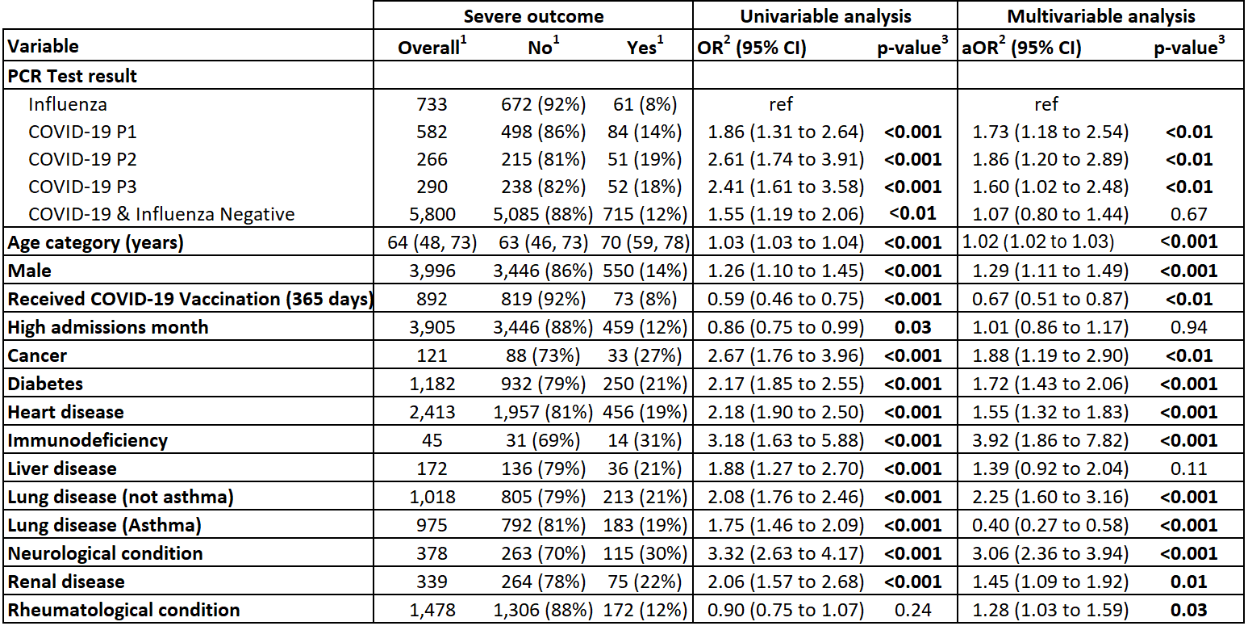


#### Supplementary Table 12. Crude and adjusted odds ratios for in-hospital death with a continuous age variable, EuroSAVE, 2021-2024.


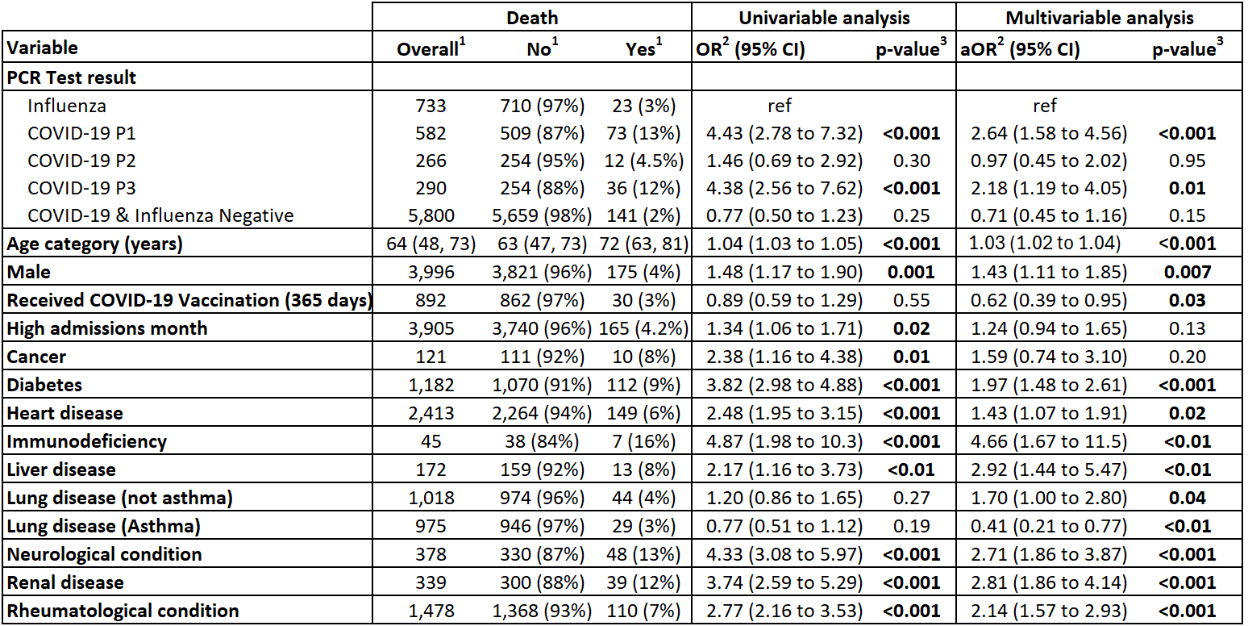


#### Supplementary Table 13. Crude and adjusted odds ratios for in-hospital severe outcomes using a two level nested model with hospital as a mixed effects variable and country as a fixed effects variable and one variable to represent the presence of any comorbidity, EuroSAVE, 2021-2024.


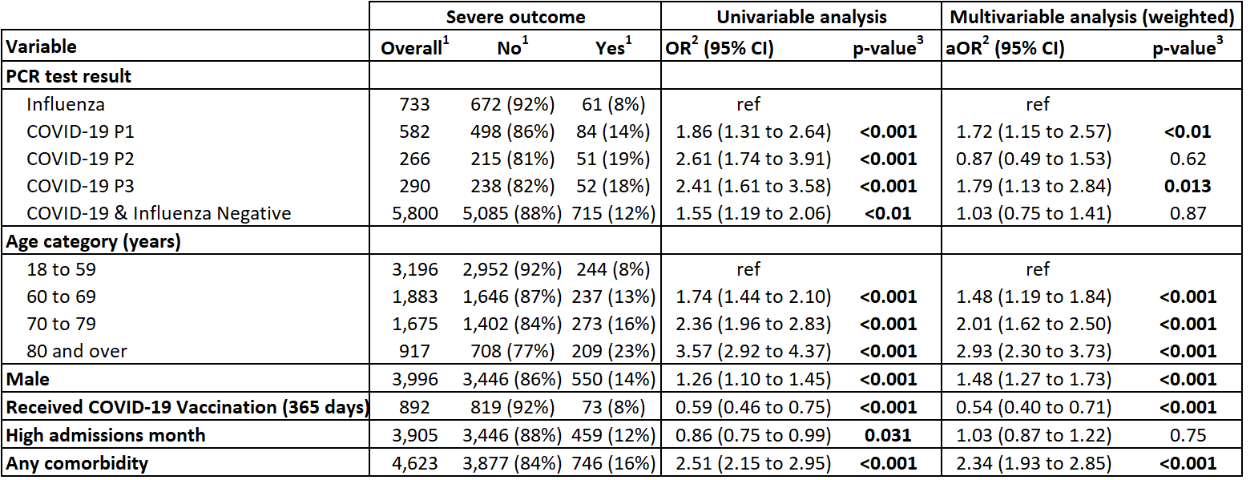


#### Supplementary Table 14. Crude and adjusted odds ratios for in-hospital death using a two level nested model with hospital as a mixed effects variable and country as a fixed effects variable and one variable to represent the presence of any comorbidity, EuroSAVE, 2021-2024.


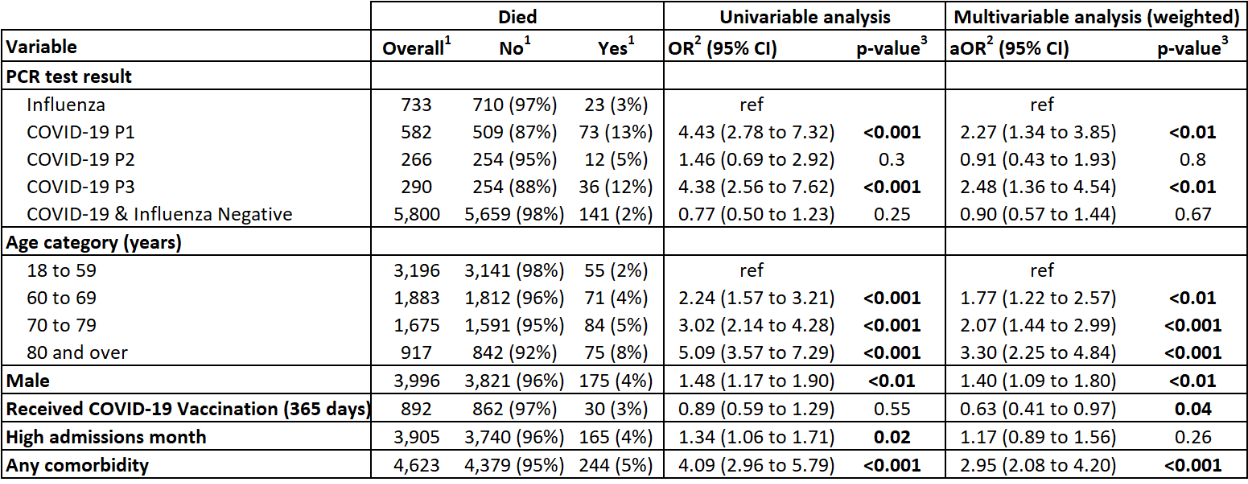


#### Supplementary Table 15. Crude and adjusted odds ratios for in-hospital death, restricted to patients who were unvaccinated for the current influenza season and for COVID-19 within the 365 days preceding onset of symptoms, EuroSAVE, 2021-2024.


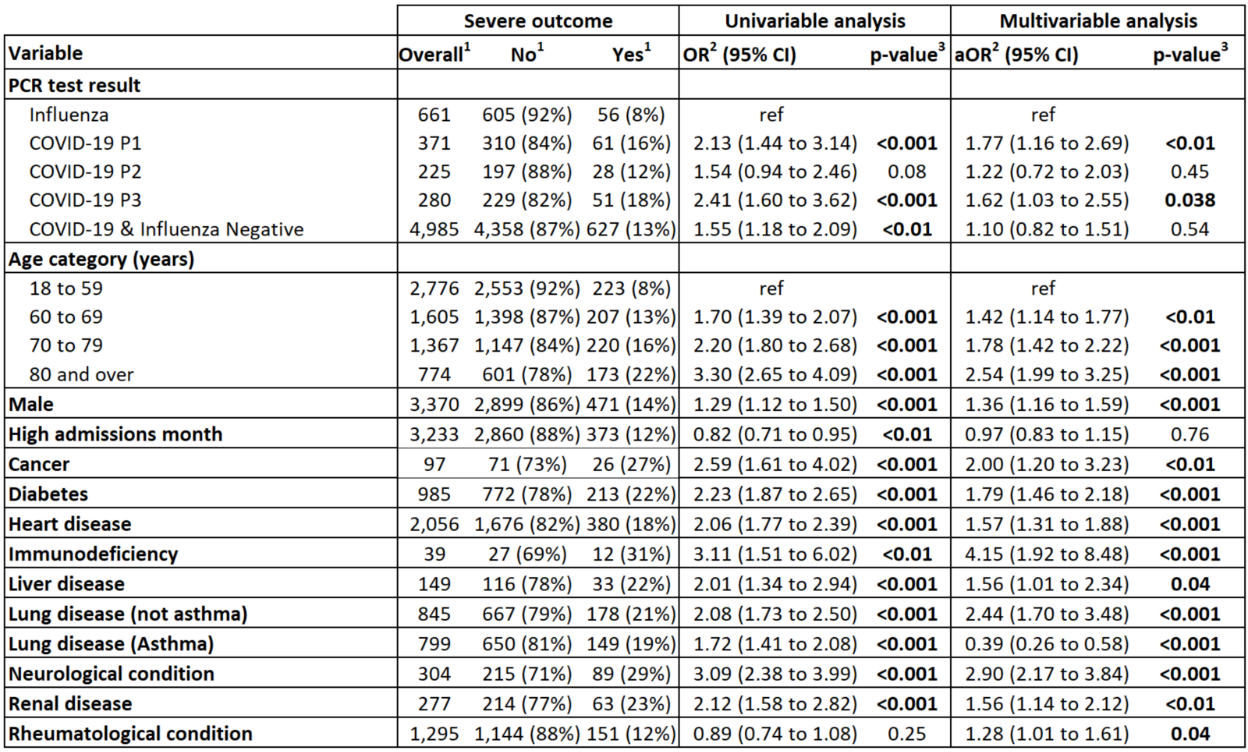


#### Supplementary Table 16. Crude and adjusted odds ratios for in-hospital severe outcomes, restricted to patients who were unvaccinated for the current influenza season and for COVID-19 within the 365 days preceding onset of symptoms, EuroSAVE, 2021-2024.


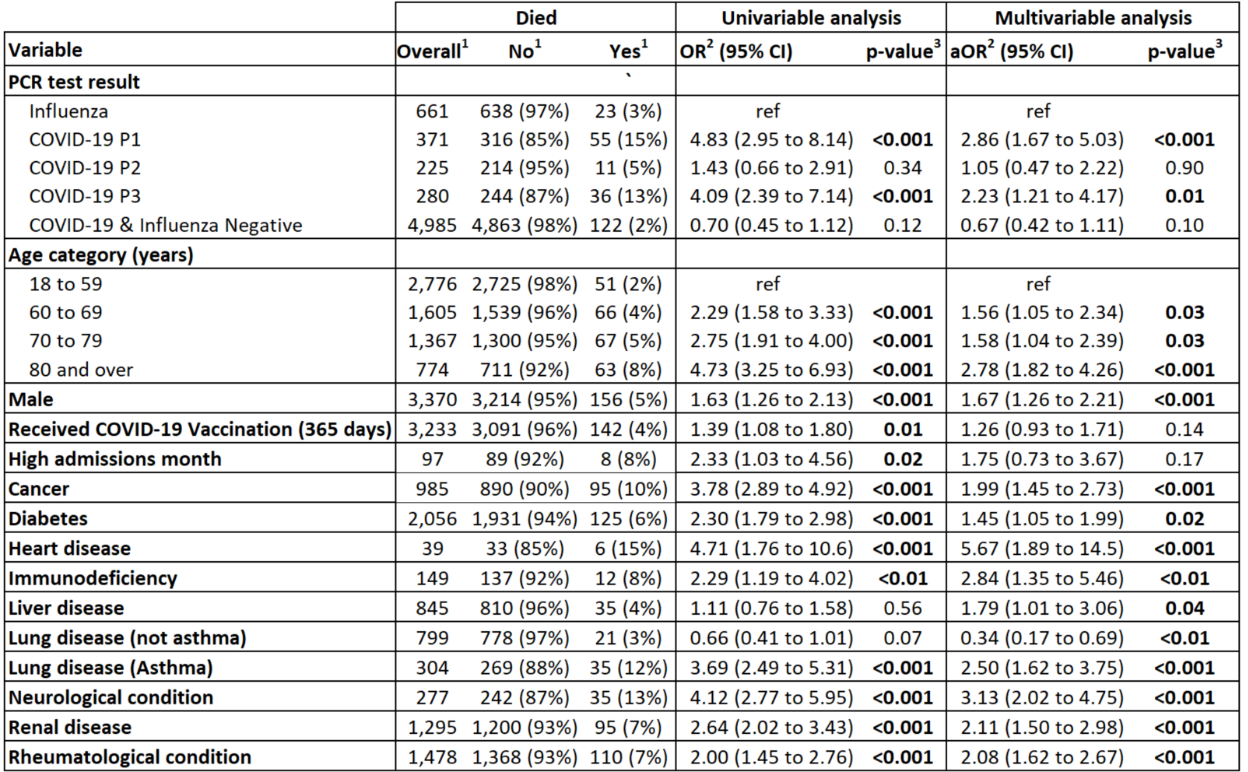


#### Supplementary Table 17. Crude and adjusted odds ratios for in-hospital severe outcomesusing inverse probability weighting with propensity scores calculated for all covariables across the five PCR test result categories and one variable to represent the presence of any comorbidity, EuroSAVE, 2021-2024.


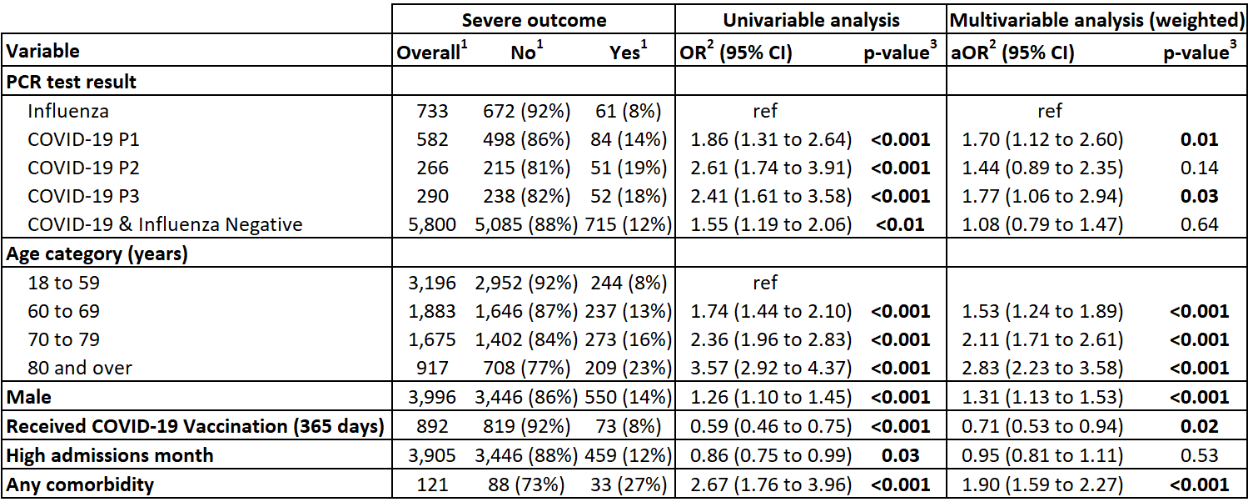


#### Supplementary Table 18. Crude and adjusted odds ratios for in-hospital death, using inverse probability weighting with propensity scores calculated for all covariables across the five PCR test result categories and one variable to represent the presence of any comorbidity, EuroSAVE, 2021-2024.


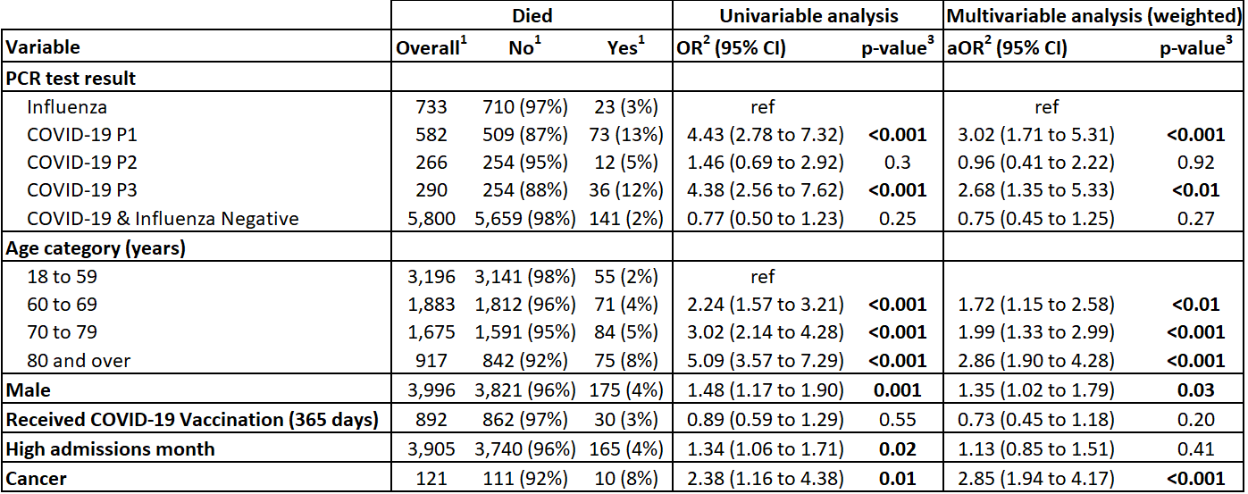


#### Supplementary Table 19. Crude and adjusted odds ratios for in-hospital severe outcome with influenza-positive patients restricted to those with influenza A only, EuroSAVE, 2021-2024.


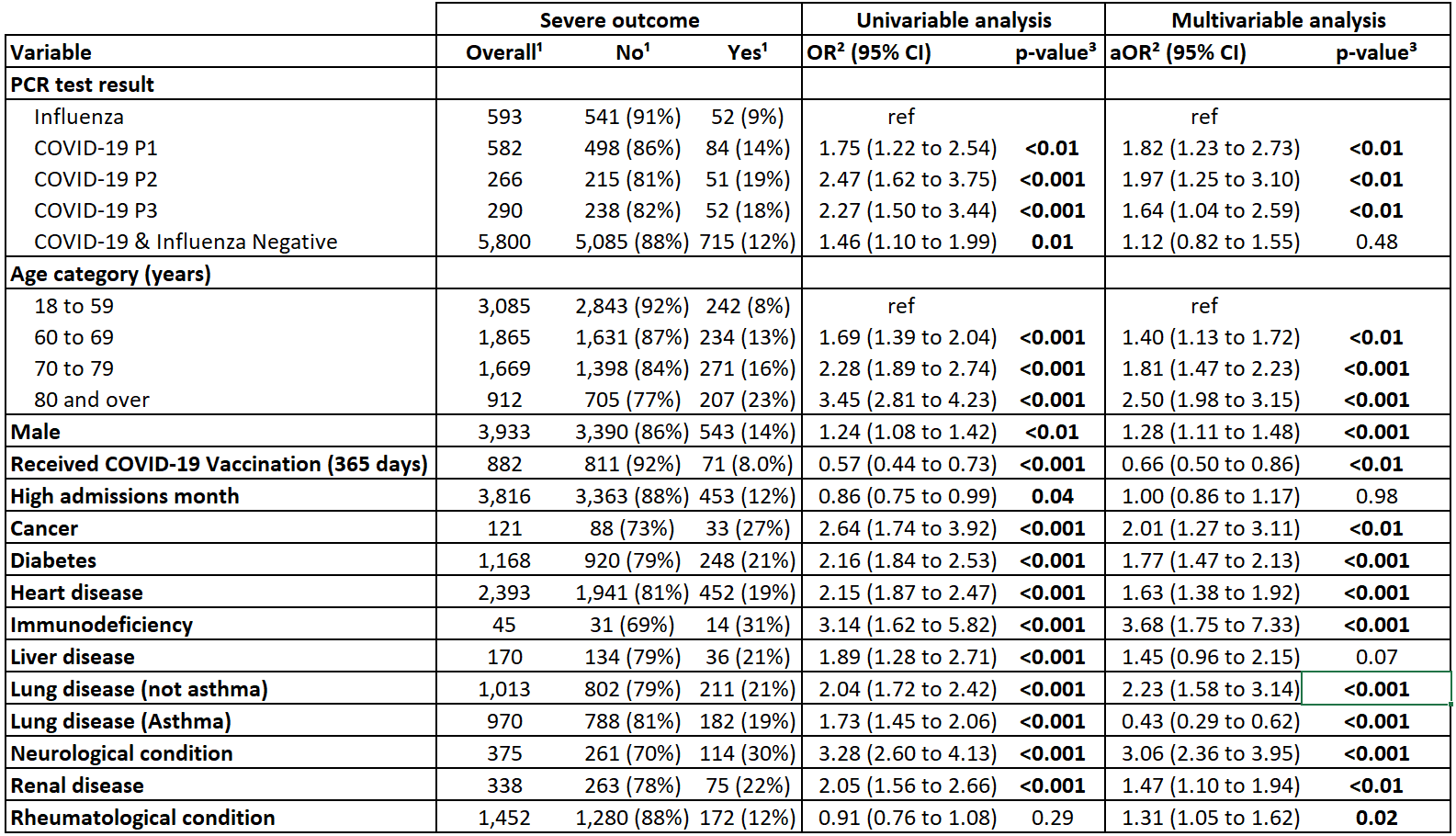


#### Supplementary Table 20. Crude and adjusted odds ratios for in-hospital death with influenza-positive patients restricted to those with influenza A only, EuroSAVE, 2021-2024.


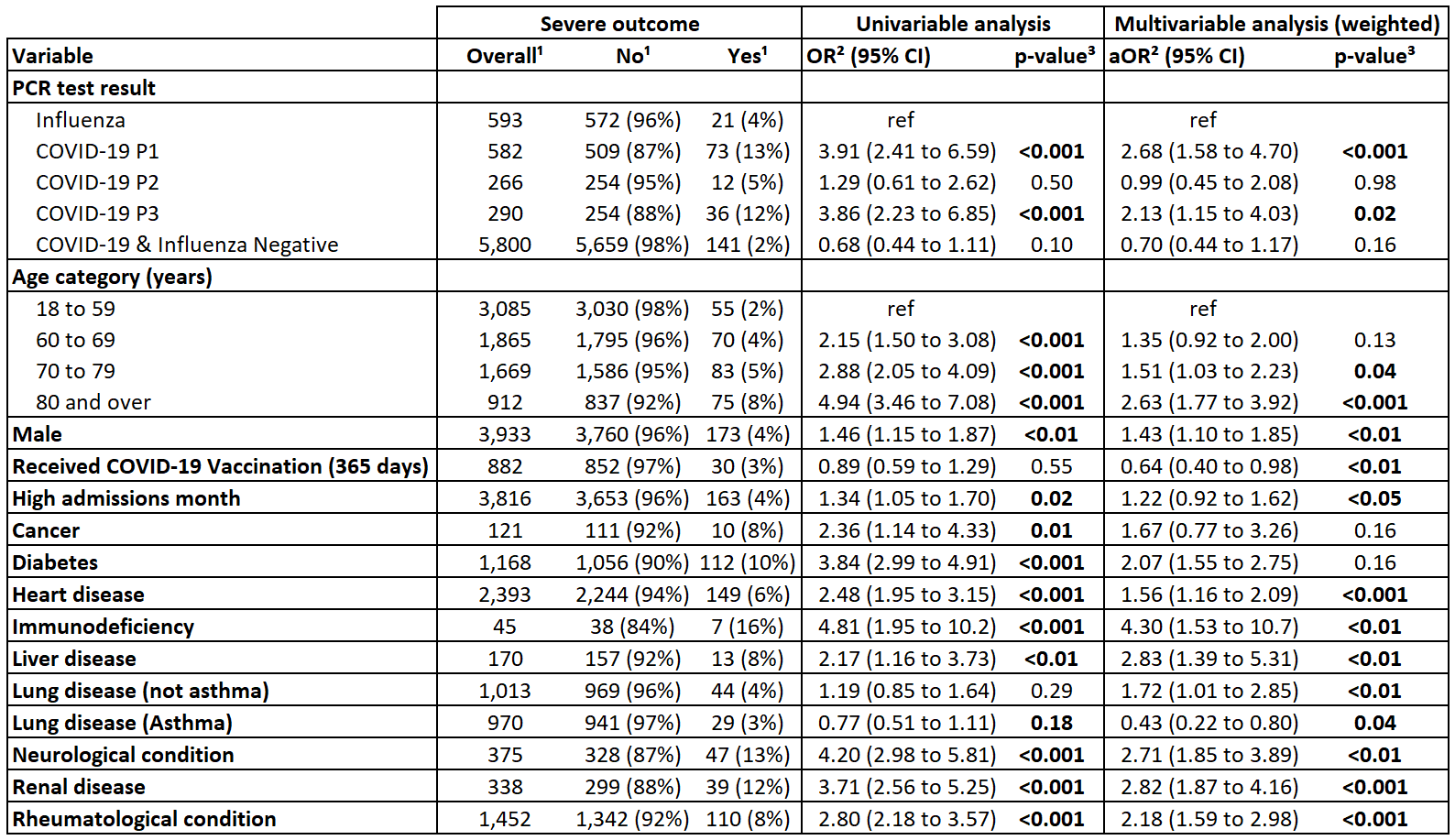


#### Supplementary Table 21. Crude and adjusted odds ratios for in-hospital severe outcome with influenza-positive patients restricted to those with influenza A/H1 only, EuroSAVE, 2021-2024.

*
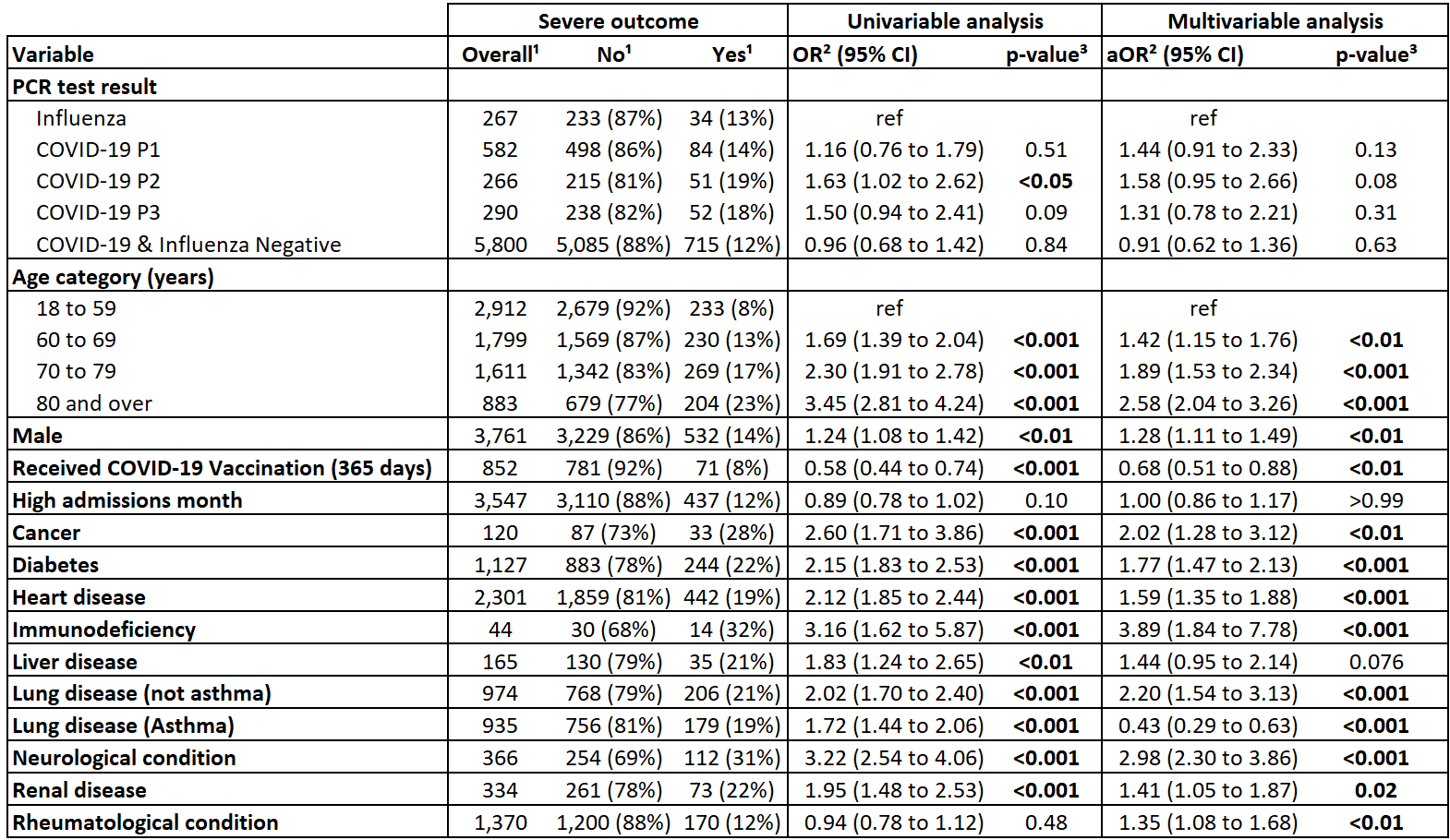
*

#### Supplementary Table 22. Crude and adjusted odds ratios for in-hospital death with influenza-positive patients restricted to those with samples identified as influenza subtype A/H1, EuroSAVE, 2021-2024.


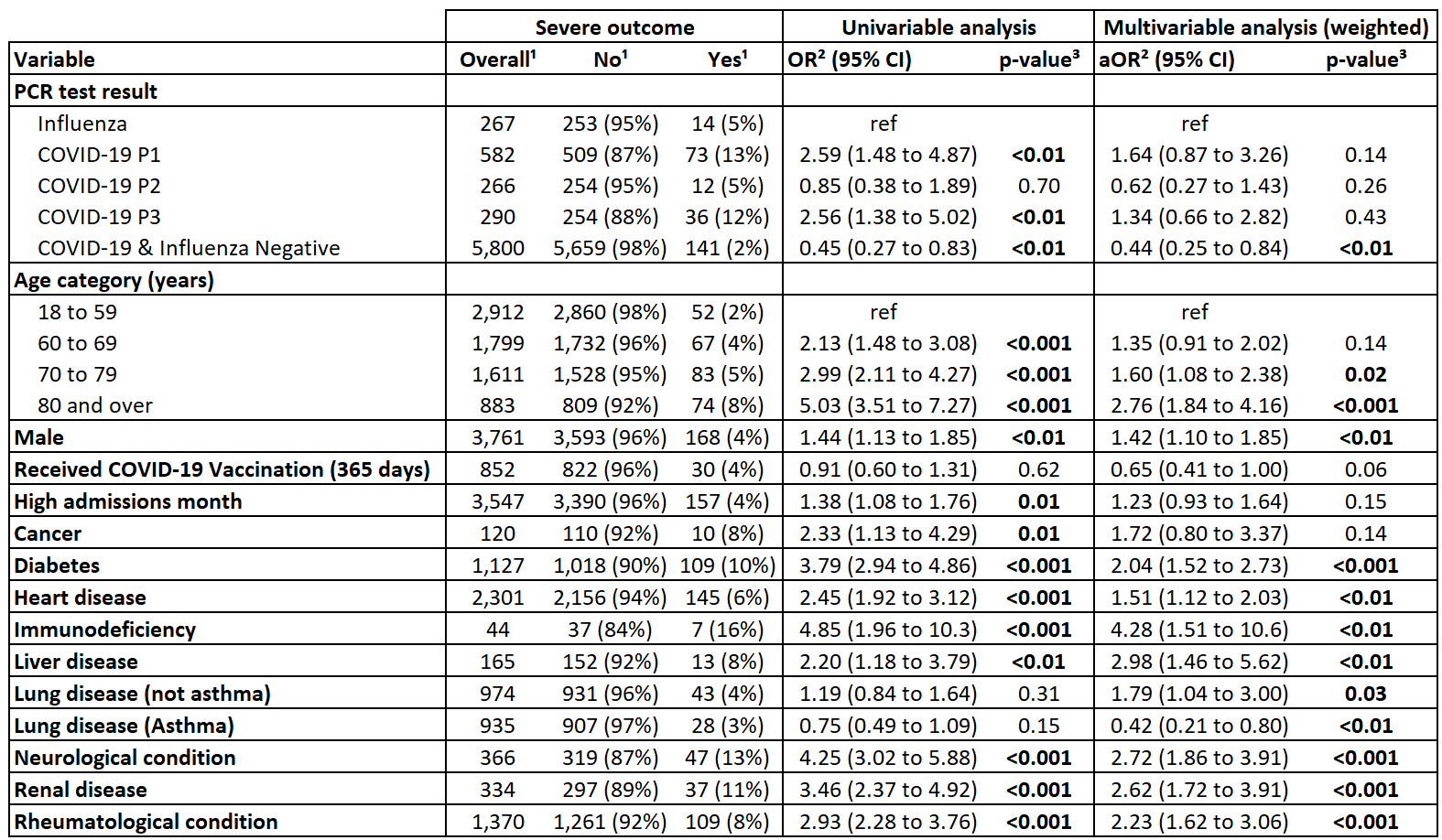


#### Supplementary Table 23. Study variables, types, descriptions, and values.
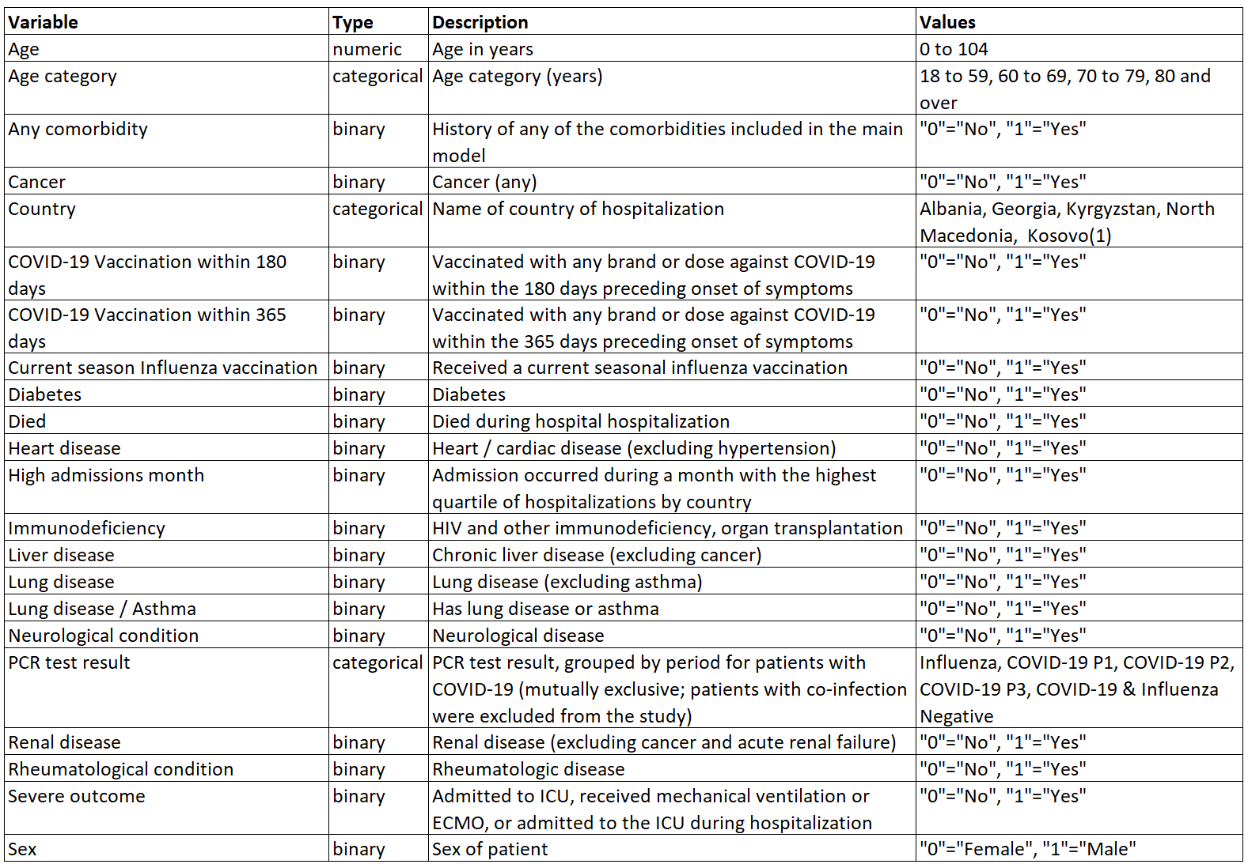

Supplement: Supplementary Tables S1–S23 [file mmc1.docx]
